# PSGL-1 decorated with sialyl Lewis^a/x^ promotes high affinity binding of myeloma cells to P-selectin but is dispensable for E-selectin engagement

**DOI:** 10.1038/s41598-024-52212-2

**Published:** 2024-01-19

**Authors:** Michael O’Dwyer, Lucy Kirkham-McCarthy, Marina Cerreto, Robin Foà, Alessandro Natoni

**Affiliations:** 1 Translational Research Facility, University of Galway, Galway, Ireland; 2 Biomedical Sciences, School of Medicine, National University of Ireland Galway, Galway, Ireland; 3 Hematology, Department of Translational and Precision Medicine, Sapienza University, Rome, Italy

**Keywords:** Metastasis, Cancer, Cancer microenvironment, Cell biology, Cell adhesion, Cell migration, Glycobiology, Biochemistry, Glycobiology

## Abstract

Dissemination of multiple myeloma into the bone marrow proceeds through sequential steps mediated by a variety of adhesion molecules and chemokines that eventually results in the extravasation of malignant plasma cells into this protective niche. Selectins are a class of C-type lectins that recognize carbohydrate structures exposed on blood borne cells and participate in the first step of the extravasation cascade, serving as brakes to slow down circulating cells enabling them to establish firm adhesion onto the endothelium. Myeloma cells enriched for the expression of selectin ligands present an aggressive disease in vivo that is refractory to bortezomib treatment and can be reverted by small molecules targeting E-selectin. In this study, we have defined the molecular determinants of the selectin ligands expressed on myeloma cells. We show that PSGL-1 is the main protein carrier of sialyl Lewis^a/x^-related structures in myeloma. PSGL-1 decorated with sialyl Lewis^a/x^ is essential for P-selectin binding but dispensable for E-selectin binding. Moreover, sialylation is required for E-selectin engagement whereas high affinity binding to P-selectin occurs even in the absence of sialic acid. This study provides further knowledge on the biology of selectin ligands in myeloma, opening the way to their clinical application as diagnostic tools and therapeutic targets.

## Introduction

Extravasation of blood borne cells such as leukocytes and hematopoietic stem cells (HSC) consists of a series of tightly regulated processes whereby cells migrate from the blood stream into target tissues by crossing the vascular endothelium^1,2^. The first step of the extravasation cascade is the slow tethering and rolling of emigrating cells onto the endothelium that is mediated by selectins^3^. These are C-type lectins that bind carbohydrate structures present on glycoproteins and glycolipids in a calcium-dependent manner^4,5^. Three types of selectins have been described so far. L-selectin is present on the surface on all leukocytes, P-selectin is expressed by endothelial cells and platelets upon activation whereas E-selectin is constitutively expressed within the microvessels in the bone marrow (BM) and skin. Expression of E-selectin can also occur in the endothelium of inflamed tissues following pro-inflammatory stimuli such as tumor necrosis factor (TNF), interleukin 1β (IL-1β) and lipopolysaccharide (LPS)^6,7^. The canonical carbohydrates recognized by all selectins are the tetrasaccharide sialyl Lewis x (SLe^x^), also known as CD15s, and its isomer sialyl Lewis a (SLe^a^). However, selectins can recognize glycosylated structures not directly related to SLe^a/x^ such as non-sialylated glycosphingolipids (sulfatides)^8,9^, CD24^10^, heparin and heparan sulphate^11,12^. SLe^a/x^ is presented on the cell surface by a vast array of proteins and lipids that form the scaffolds of the glycoconjugates known as selectin ligands. Both SLe^a/x^ and its scaffolds participate in the formation of the molecular interface that is recognized by the selectins, contributing to the specificity and the affinity of selectin binding, which can also be regulated by additional modifications such as sulphation^13^. For instance, P-selectin glycoprotein ligand-1 (PSGL-1) when decorated with SLe^a/x^ can bind all three types of selectins; however, the specificity toward a particular one is defined by sulphation^14^. Indeed, binding to L-selectin requires sulphation of the N-acetylglucosamine (GlcNAc) in the SLe^x^ backbone (6 sulfo-SLe^x^) whereas sulphation of critical tyrosine residues (Tyr-46, Tyr-48, Tyr-51) in the protein scaffold is essential for P-selectin binding; lastly, PSGL-1 binding to E-selectin is independent of sulphation^14^.

Although selectins participate in the physiological recruitment of leukocytes into secondary lymphoid tissues and sites of inflammation, it is now clear that selectins and their ligands are also involved in cancer dissemination and metastasis^15,16^. Indeed, high levels of SLe^a/x^ have been correlated with poor prognosis and metastatic disease in several tumors^15,17–19^. Selectin ligands contribute to cancer metastasis by different means, such as increasing cell adhesion and rolling on endothelial cells of target organs and promoting cancer cell survival in the blood stream by recruiting platelets on the tumor cell surface^15,16,19^. Of note, cancer cells may co-express on the cell surface multiple types of selectin ligands with redundant or specific functions. For instance, in acute myeloid leukemia (AML) the selectin ligands CD43, CD44 and PSGL-1 all contribute to E-selectin binding^20^; however, only PSGL-1 seems to be involved in E-selectin-mediated chemoresistance of AML blasts^21^.

Multiple myeloma (MM) is a plasma cell malignancy characterized by the abnormal proliferation and accumulation of plasma cells in the BM^22^. MM cells are rather vulnerable in the circulation where they are subjected to high shear stress and immune cell attack. Thus, MM cells have evolved mechanisms that allow them to survive in the circulation and to quickly home to the BM, which represents a niche where MM cells are nurtured and protected from chemotherapeutic drugs^23,24^. In this respect, we have previously shown that MM cells enriched for the expression of selectin ligands display an aggressive phenotype characterized by a complete resistance to bortezomib in vivo, which can be reverted by blocking E-selectin with small glycomimetic molecules or by inhibiting sialyltransferases, which are involve in the generation of selectin ligands^25,26^. Moreover, these cells strongly bind to P-selectin in vitro leading to the establishment of specific interactions with platelets that protect them from natural killer (NK) cell-mediated cytotoxicity^27^.

In an attempt to characterize putative selectin ligands expressed on MM, we found PSGL-1 to be the predominant protein ligand. PSGL-1 carries SLe^a/x^ only in MM cells that are enriched for the expression of selectin ligands and is required for P-selectin binding. However, PSGL-1 decorated with SLe^a/x^ exhibits a redundant role for E-selectin engagement. We also showed that sialylation of MM cells is essential for E-selectin but not for P-selectin engagement, suggesting that in MM sialylation of PSGL-1 is not an absolute requirement for P-selectin binding. Our work defines the molecular determinants of selectin binding in MM with important clinical implications for the development of novel therapies targeting PSGL-1 and other selectin ligands.

## Results

### Identification of putative E-selectin protein ligands on MM cells by mass spectrometry

We have previously shown that the Heca452 antibody recognizes a subpopulation of myeloma cells that are enriched for the expression of SLe^a/x^, which display strong E-selectin binding activity and resistance to bortezomib in vivo^25,26^. Heca452 is a rat monoclonal antibody that was originally developed to detect antigens expressed on high endothelial venules of lymphoid organs^28^. Subsequent studies revealed that Heca452 is a non-blocking antibody that recognizes neuraminidase-sensitive SLe^a/x^ structures^29,30^. To identify and characterize the putative E-selectin ligands detected by the Heca452 antibody, we first isolated membrane enriched fractions derived from the MM1S Heca452 enriched (MM1S^Heca452^) and parental (MM1S^Parental^) cell lines and then performed an immunoprecipitation (IP) experiment using the Heca452 or matched-isotype antibody followed by liquid chromatography–mass spectrometry (LC–MS) on the eluted immuno-complexes. The results obtained from the LC–MS were filtered so that all the proteins identified by less than two peptides were not considered for the subsequent analysis. After this first criterion, proteins that were immunoprecipitated by the isotype antibody were excluded. Using this strategy, we identified 774 proteins in the MM1S^Heca452^ and 13 proteins in the MM1S^Parental^ sample respectively (Supplementary Table S1 and S2). The results were listed in ascending order according to the area parameter value. P-selectin glycoprotein ligand-1 (PSGL-1; accession number: B4DHR9) represented the top ranked protein in the MM1S^Heca452^ sample (area value: 6.1 × 10^8^; peptides: 6). PSGL-1 was also identified in the MM1S^Parental^ sample albeit the area value and the number of unique peptides were lower (area value: 6.8 × 10^5^; peptides: 2) than in the MM1S^Heca452^ sample, suggesting an enrichment of PSGL-1 in the latter. Of note, similar results were obtained when the experiment was repeated in the RPMI8226^Heca452^ and RPMI8226^Parental^ cell lines (Supplementary Table S3 and S4).

### PSGL-1 is modified by SLe^a/x^ in the Heca452 enriched MM cells

**Figure 1:**
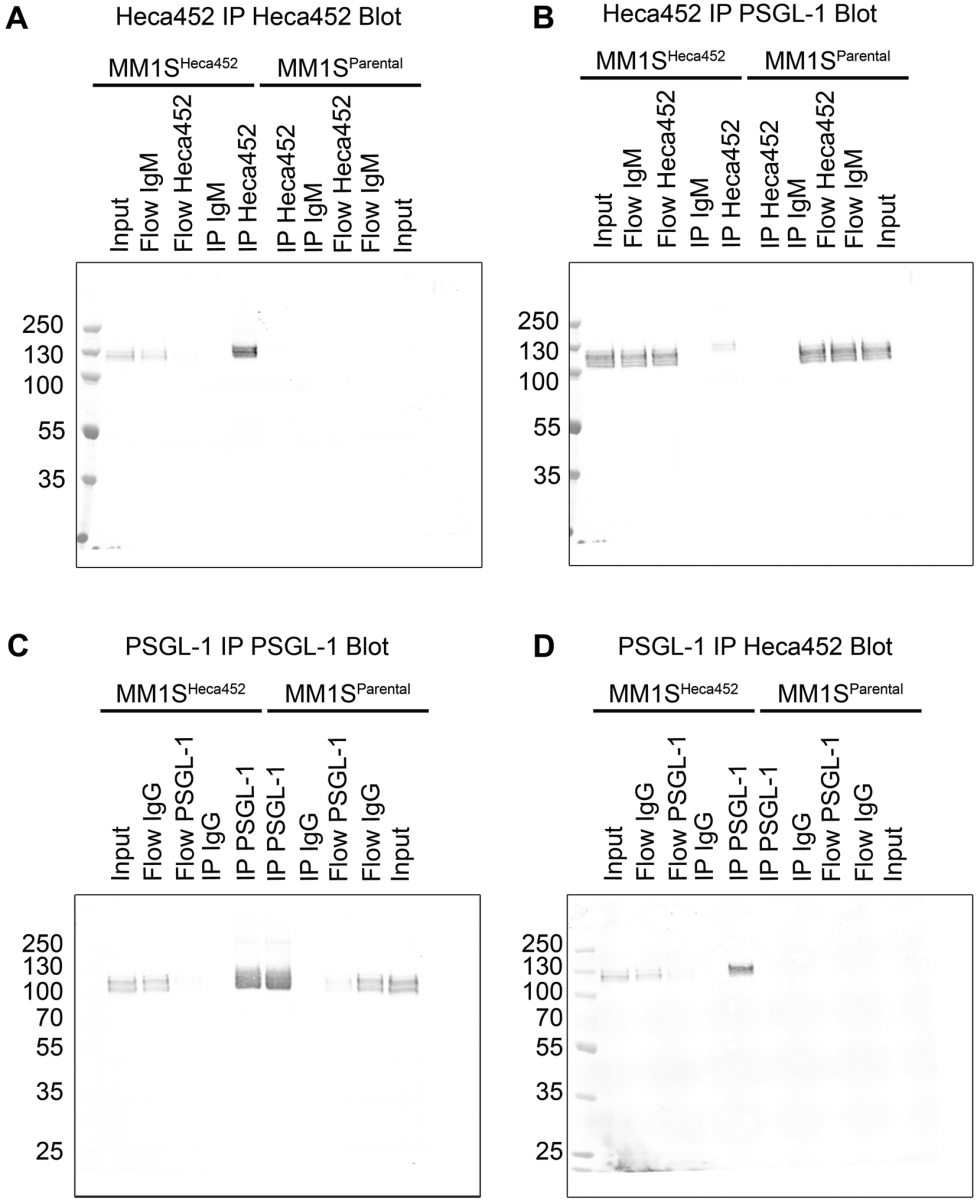
PSGL-1 is a carrier for SLe^a/x^-related structures in the MM1S^Heca452^ cells. Membrane-enriched fractions from the MM1S^Heca452^ and the MM1S^Parental^ cells were subjected to an immunoprecipitation using the Heca452 (**A**,**B**) or PSGL-1 (**C**,**D**) antibodies and their matched isotype controls. The eluted protein complexes were run on an SDS-PAGE, transferred to a nitrocellulose membrane and blotted for Heca452 (**A**,**D**) or PSGL-1 (**B**,**C**). Labels above the blots represent the different samples. Numbers on the left hand side represent the molecular weight marker. Western blot analysis depicted in the figure is representative of 2 independent experiments.

We next performed a series of IP experiments followed by Western blot analysis to confirm the LC–MS data. First, we carried out an IP on the MM1S^Heca452^ and MM1S^Parental^ membrane enriched extracts using the Heca452 or matched-isotype antibody and analyzed the eluted proteins by Western blot. The Heca452 IP was rather efficient and specific since almost all the proteins were depleted from the input by the Heca452 antibody but not by the matched-isotype control (Fig. 1A). Of note, the Heca452 signal was detected only in the MM1S^Heca452^ cells with a prominent band localized near the 130 kDa molecular weight (MW) marker, suggesting that only a few proteins with an apparent molecular weight of 130 kDa carry the SLe^a/x^ modification. Importantly, no signal was detected in the matched-isotype IP. We then analyzed the Heca452 IP by Western blot using an anti-PSGL-1 antibody. PSGL-1 was detected in the input of the MM1S^Heca452^ and MM1S^Parental^ lines and appeared as multiple bands spanning from the 100 to 130 kDa MW markers, indicating a high degree of modifications (Fig. 1B). Importantly, PSGL-1 was detected in the IP sample as a 130 kDa MW band in the MM1S^Heca452^ but not in the MM1S^Parental^ sample, indicating that PSGL-1 is the major 130 kDa MW band detected by the Heca452 antibody. Moreover, the Heca452 IP could not deplete all the PSGL-1 present in the MM1S^Heca452^ input since it was still observed in the Flow sample. This evidence, together with the difference in the apparent MW of PSGL-1 in the input and IP samples, suggests that only a fraction of PSGL-1 is modified by the SLe^a/x^. We then performed the reciprocal IP where we immunoprecipitated PSGL-1 and examined the eluted proteins by Western blot using an anti-PSGL-1 and the Heca452 antibody. PSGL-1 was efficiently and specifically immunoprecipitated from both the MM1S^Heca452^ and MM1S^Parental^ cells (compare Input and Flow lanes; Fig. 1C). In addition to the main band of about 100–130 kDa, we could detect an additional band in the IP samples running above 250 kDa MW, which probably represents the dimeric form of PSGL-1. Despite efficient immunoprecipitation of PSGL-1 in both MM1S^Heca452^ and MM1S^Parental^ cells, the Heca542 band was detected only in the MM1S^Heca452^ line with an apparent MW of 130 kDa (Fig. 1D). Of note, the Heca452 signal was almost completely depleted from the MM1S^Heca452^ input by the PSGL-1 IP. Altogether, these data demonstrate that indeed PSGL-1 carries the SLe^a/x^ modification primarily in the MM1S^Heca452^ cells, suggesting an enrichment of the SLe^a/x^ modified PSGL-1 in the Heca452 enriched cells.

### Knock down of PSGL-1 did not inhibit the interactions between MM cells and E-selectin

**Figure 2:**
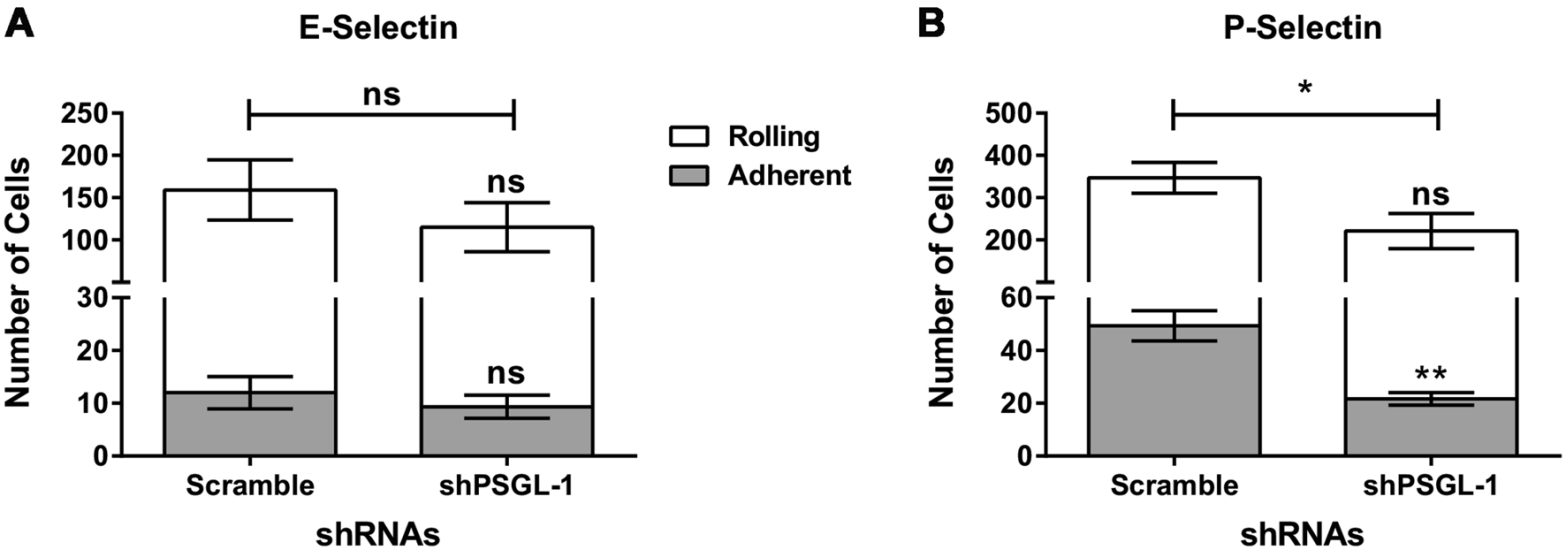
Downregulation of PSGL-1 inhibits rolling and adhesion on P-selectin but not on E-selectin. MM1S^Heca452^ cells stably expressing a scramble shRNA control or the shRNA for PSGL-1 were tested in a rolling and adhesion assay under shear stress on recombinant E-selectin (**A**) and P-selectin (**B**), both at 15 µg/ml. Histograms represent the mean + sem of three independent experiments performed in duplicate. Symbols on top of the error bars represent the statistical significance of the rolling (white) or adhesion (grey) fractions compared to the same fractions in the scramble control. Symbols on top of the horizontal bars represent the statistical significance of the total fraction (rolling plus adhesion fraction). The unpaired one-tailed t test was used to determine statistical significance. **p < 0.01; *p < 0.05; ns non-significant.

We next wanted to assess whether PSGL-1 serves as an E-selectin ligand in MM cells. To this end, we knocked down PSGL-1 in the MM1S^Heca452^ enriched cells using two shRNAs for PSGL-1 and tested the knockdown cells in a rolling assay under shear stress on E-selectin. We also included P-selectin in the assay as PSGL-1 is known to be the major P-selectin ligand in MM^31^ and we have recently reported that Heca452 enriched cells displayed a higher P-selectin binding activity compared to that of the parental lines^27^. Only one shRNA efficiently knocked down PSGL-1 as shown by Western blot (Fig. S1A, S1C). Interestingly, in the PSGL-1 knockdown cells, we observed a concomitant reduction in the levels of SLe^a/x^ by Western blot analysis (Fig. S1B, S1D), which, together with the LC–MS data, indicates that PSGL-1 could be the major protein scaffold of the SLe^a/x^. Most of the scramble MM1S^Heca452^ cells displayed rolling on E-selectin and only few adherent cells, as previously reported (Fig. 2A)^25^. PSGL-1 knockdown did not decrease significantly the rolling nor the adherent cells on E-selectin (Fig. 2A). However, the knockdown of PSGL-1 did induce a slight increase in the velocity of rolling cells, which indicates that under conditions where the PSGL-1 levels are reduced, MM cells establish weaker bindings with E-selectin (Fig. S2A). On the contrary, the knockdown of PSGL-1 diminished the number of cells interacting with P-selectin, mainly due to a reduction of the adherent fraction (Fig. 2B). We could also observe a mild increase in the rolling velocity (Fig. S2B).

### Blocking of PSGL-1 decreases the number of MM cells binding to P-selectin but not to E-selectin

**Figure 3:**
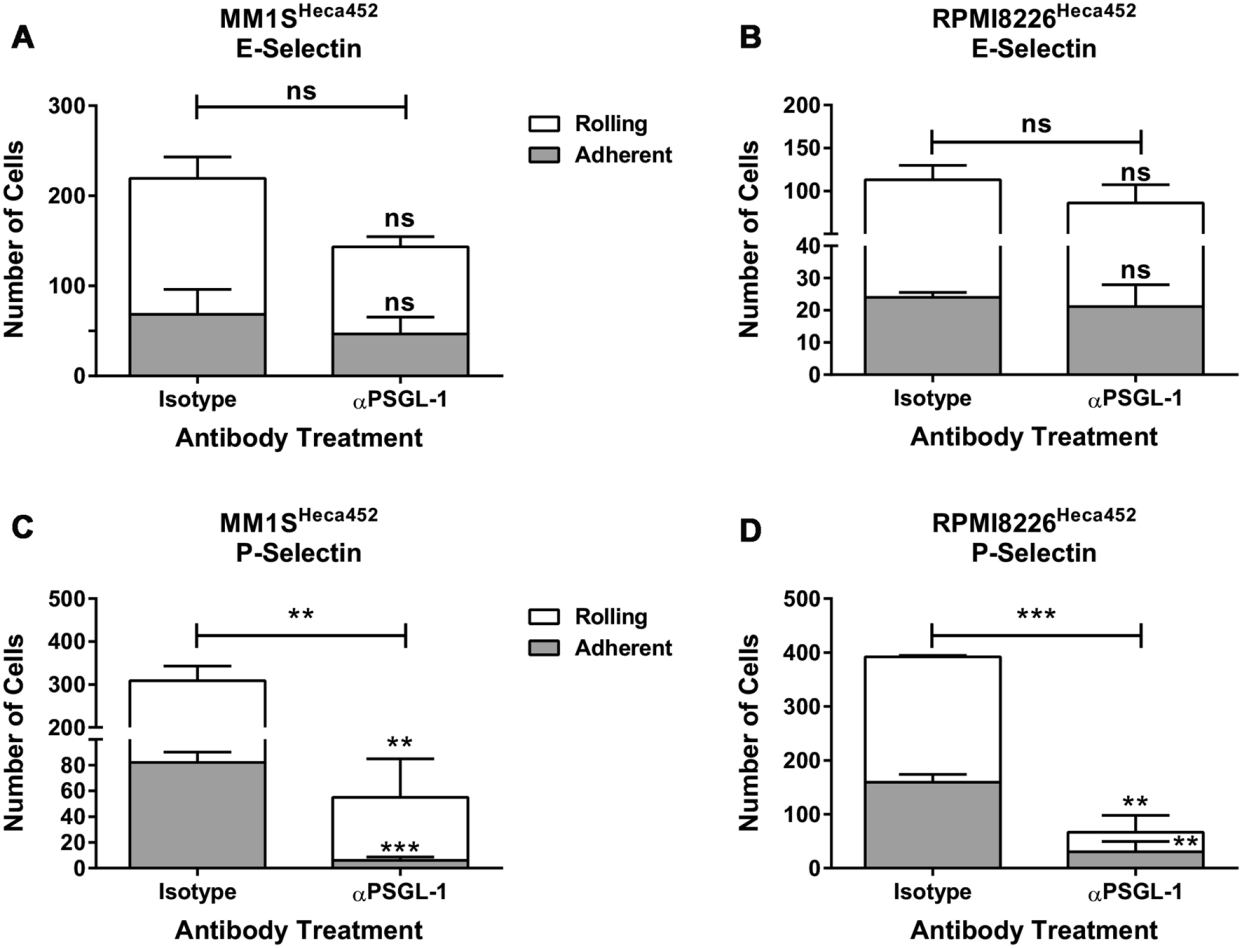
PSGL-1 neutralization with a blocking antibody inhibits rolling and adhesion on P-selectin but not on E-selectin. MM1S^Heca452^ (**A**,**C**) and RPMI8226^Heca452^ (**B**,**D**) cells were pre-incubated for 1 h with 10 µg/ml of an anti-PSGL-1 blocking antibody or matched-isotype control and then tested in a rolling and adhesion assay under shear stress on recombinant E-selectin (**A**,**B**) and P-selectin (**C**,**D**), both at 15 µg/ml. Histograms represent the mean + sem of three independent experiments performed in duplicate. Symbols on top of the error bars represent the statistical significance of the rolling (white) or adhesion (grey) fractions compared to the same fractions in the isotype control. Symbols on top of the horizontal bars represent the statistical significance of the total fraction (rolling plus adhesion fraction). The unpaired one-tailed t test was used to determine statistical significance. ***p < 0.001; **p < 0.01; ns non-significant.

To confirm the results obtained in the PSGL-1 knockdown cells, we carried out a series of experiments using a pharmacological approach consisting in neutralizing PSGL-1 expressed on the surface of MM cells with a blocking antibody. MM1S^Heca452^ and RPMI^Heca452^ cells were incubated with an anti-PSGL-1 blocking antibody or a matched-isotype control and then tested in a rolling assay on both E- and P-selectin. The anti-PSGL-1 blocking antibody did not reduce the number of MM cells interacting with E-selectin in both cell lines (Fig. 3A,B), however we could observe a decrease in the number of rolling cells in the MM1S^Heca452^ line (Fig. 3A), although non statistically significant, and an increase in the rolling velocity of the MM1S^Heca452^ but not of the RPMI^Heca452^ cells (Fig. S3A, S3B). However, the blocking antibody greatly reduced the number of MM cells interacting with P-selectin in both cell lines, which was the result of a decrease of the rolling as well as adherent cells (Fig. 3C,D). These results suggest that MM cells express additional ligands that may compensate for PSGL-1 in the binding to E-selectin, whereas PSGL-1 is the major P-selectin ligand in Heca452 enriched MM cells.

### Pronase treatment abrogates MM binding to P-selectin but not to E-selectin

**Figure 4:**
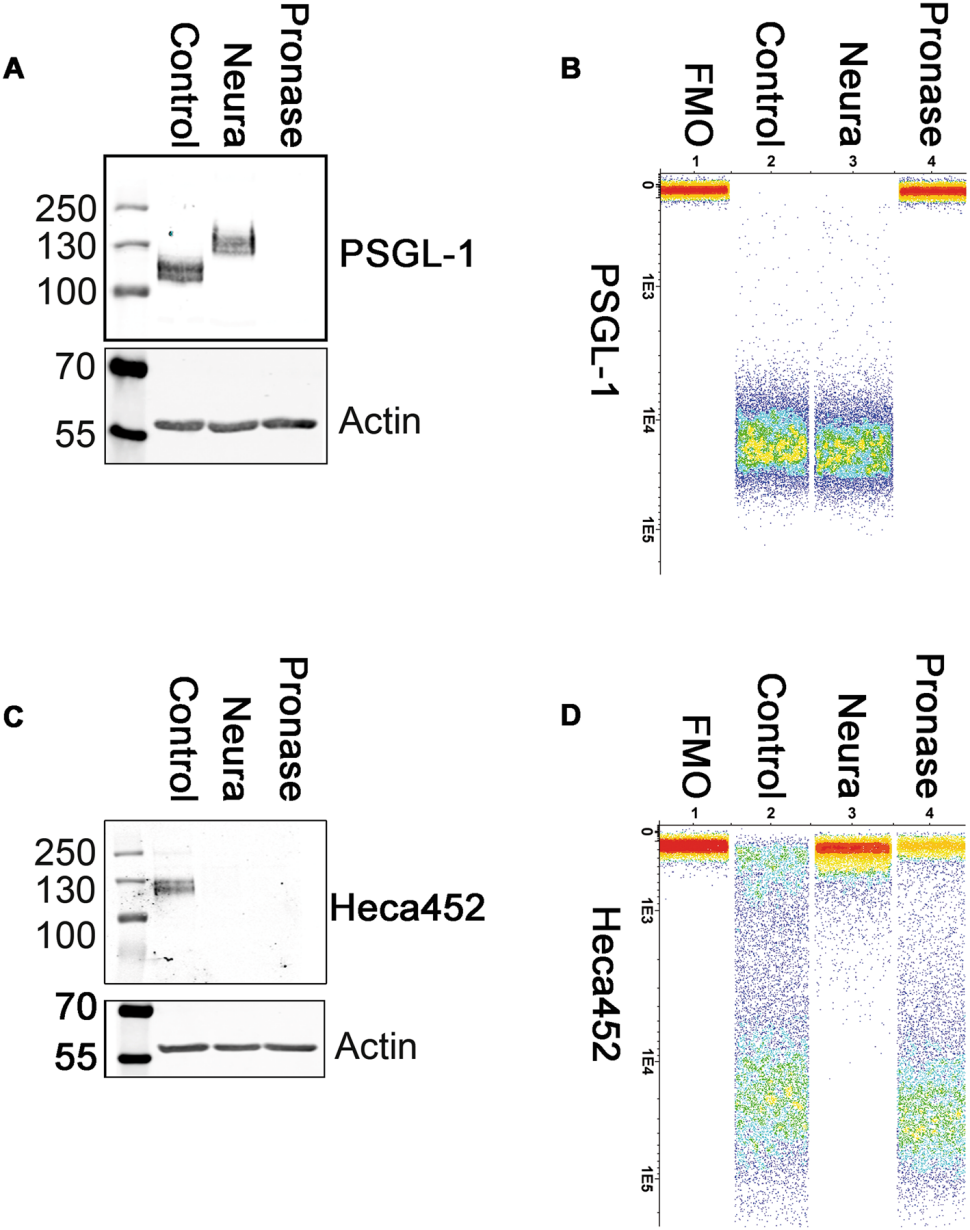
Neuraminidase and pronase treatments differentially affect the levels of PSGL-1 and Heca452 in the MM1S^Heca452^ cells. Western blot (**A**,**C**) and flow cytometry (**B**,**D**) analysis of MM1S^Heca452^ cells pre-treated/mock-treated with either neuraminidase (1 mU/ml) or pronase (1 mg/ml) for 45 min at RT. After treatment, cells were either lysed for Western blot analysis or stained with the indicated antibodies and analyzed by flow cytometry. Cell extracts were subjected to SDS-PAGE, transferred onto a nitrocellulose membrane and blotted for PSGL-1, Heca452 and β-actin, used as loading control. Labels above the blots represent the different treatments whereas labels on the right hand side of the blots indicate the antibodies used for blotting. Numbers on the left hand side of the blots represent the molecular weight marker. The flow cytometry analysis is reported as a band plot generated with the Infinicyt software v 2.0.5.b.007. Labels above the band plots represent the different treatments whereas labels on the left hand side of the band plots indicate the antibody used for the staining. The Western blot and flow cytometry analyses depicted in the figure are representative of 3 independent experiments.

**Figure 5:**
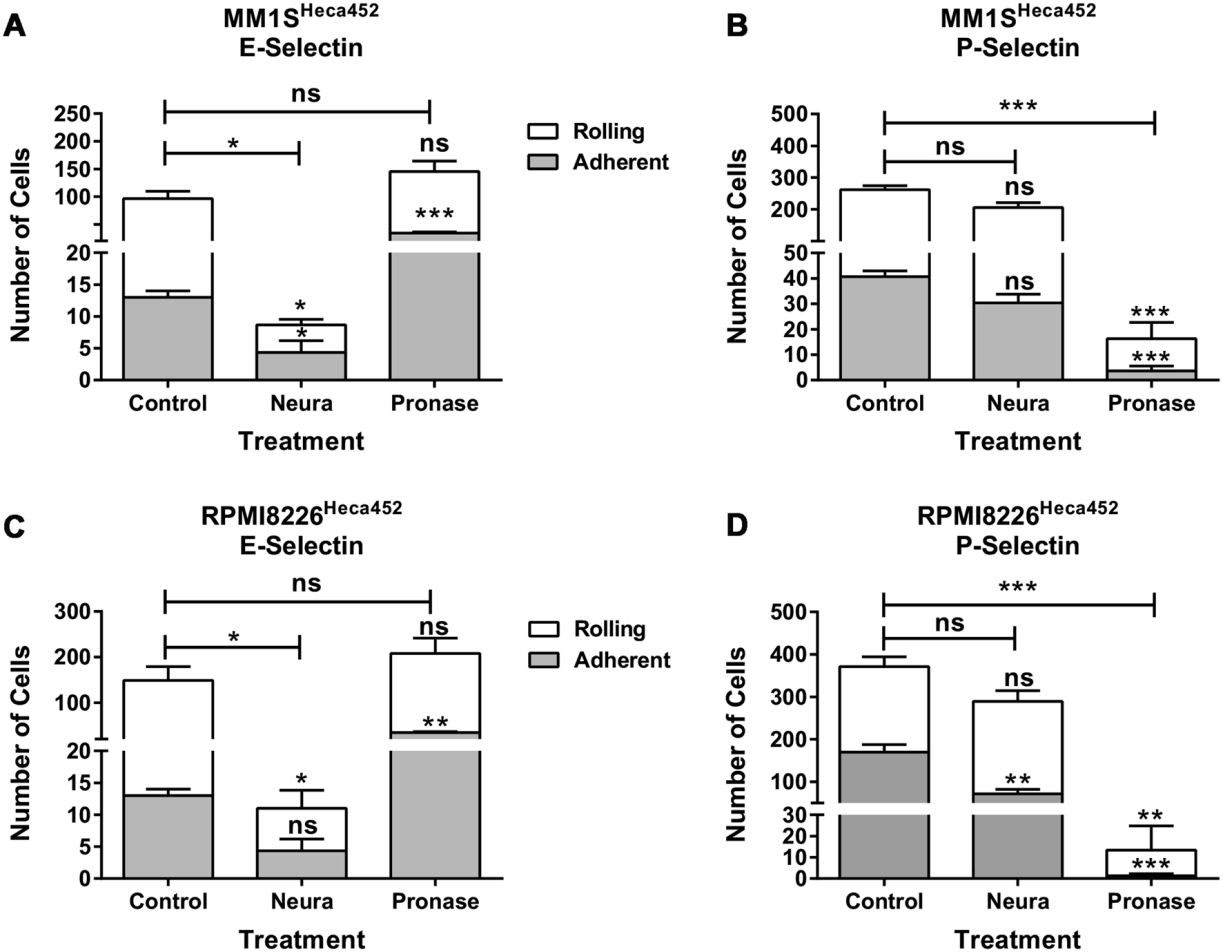
Neuraminidase and pronase treatments differentially affect rolling and adhesion under shear stress on E- and P-selectins. MM1S^Heca452^ (**A**,**B**) and RPMI8226^Heca452^ (**C**,**D**) cells were pre-treated/mock-treated with either neuraminidase (1 mU/ml) or pronase (1 mg/ml) for 45 min at RT and then tested in a rolling and adhesion assay under shear stress on recombinant E-selectin (**A**,**C**) and P-selectin (**B**,**D**), both at 15 µg/ml. Histograms represent the mean + sem of three independent experiments performed in duplicate. Symbols on top of the error bars represent the statistical significance of the rolling (white) or adhesion (grey) fractions compared to the same fractions in the control treatment. Symbols on top of the horizontal bars represent the statistical significance of the total fraction (rolling plus adhesion fraction). The two-way ANOVA followed by Sidak’s multiple comparison post-hoc testing was used to determine statistical significance. *** p < 0.001; ** p < 0.01; * p < 0.05; ns non-significant.

To assess the contribution of additional protein ligands on E-selectin binding, we employed a biochemical approach by treating MM cells with pronase, a mix of different proteinases that is commonly used to characterize the structures of oligosaccharides, which should remove all glycoproteins from the cell surface ^32^. We also included neuraminidase treatment to evaluate the role of sialic acids. MM treated/mock-treated cells were first examined by Western blot and flow cytometry to determine the Heca452 and PSGL-1 expression levels on the cell surface and then tested in a rolling assay on E- and P-selectin. After pronase treatment of MM1S^Heca452^ cells, PSGL-1 was no longer detectable by both Western blot and flow cytometry analysis (Fig. 4A,B, Fig. S4A). Neuraminidase treatment did not decrease the levels of PSGL-1 but induced a marked increase in its apparent MW on the SDS-PAGE confirming that PSGL-1 is indeed sialylated (Fig. 4A, Fig. S4A). When the samples were analyzed using the Heca452 antibody, we observed that both neuraminidase and pronase treatments removed the Heca452 signal detected by Western blot, suggesting that the SLe^a/x^ was depleted from all the protein carriers present on the cell surface (Fig. 4C, Fig. S4B). However, flow cytometry analysis of the same samples revealed that the pronase treatment only decreased but did not abolish the Heca452 signal from the cell surface, indicating that SLe^a/x^ is also displayed on lipid scaffolds (Fig. 4D). Similar results were obtained in the RPMI8226^Heca452^ cell line (Fig. S5A-D, Fig. S6A, S6B). When the sample were tested in a rolling assay, we observed that neuraminidase treatment impaired the ability of the MM1S^Heca452^ cells to roll and adhere on E-selectin (Fig. 5A). Pronase treatment did not inhibit rolling on E-selectin but instead induced a significant increase in the number of adherent cells (Fig. 5A) and a concomitant decrease of the rolling velocity (Fig. S7A). These data suggest that E-selectin ligands are displayed on proteins as well as lipids, corroborating the results obtained by the Western blot and flow cytometry analysis. Interestingly, neuraminidase treatment did not inhibit rolling and adhesion of the MM1S^Heca452^ cells on P-selectin (Fig. 5B), although it did increase the rolling velocity (Fig. S7B), suggesting that, in these conditions, sialylation is dispensable for P-selectin binding. On the contrary, pronase treatment greatly reduced rolling and adhesion of MM1S^Heca452^ cells on P-selectin, confirming that protein ligands are indeed the main counter-receptors for P-selectin (Fig. 5B). Similarly, neuraminidase treatment of RPMI8226^Heca452^ cells inhibited rolling on E-selectin (Fig. 5C), whereas pronase treatment induced an increase in the number of adherent cells (Fig. 5C) as well as a decrease in the rolling velocity on E-selectin (Fig. S7C), confirming the data obtained in MM1S^Heca452^ cells. When tested on P-selectin, neuraminidase treatment did not inhibit cell rolling but induced a slightly, although significant, decrease in cell adhesion under shear stress, indicating that in the RPMI8226^Heca452^ cells sialylation may have a more important role in P-selectin binding than in MM1S^Heca452^ cells, although it should be noted that the total number of interacting cells was not significantly different between the control and the neuraminidase treated sample (Fig. 5D). Neuraminidase treatment also increased the rolling velocity of the RPMI8226 ^Heca452^ cells on P-selectin as observed for the MM1S ^Heca452^ cells (Fig. S7D). On the contrary, pronase treatment severely inhibited rolling and adhesion of RPMI8226^Heca452^ cells on P-selectin, corroborating the essential role of PSGL-1 in P-selectin binding (Fig. 5D).

## Discussion

Many years of intense investigation have acknowledged the fundamental role of the tumor microenvironment (TME) in leukemia development and progression^33^. Indeed, the reciprocal crosstalk between leukemic cells and the TME establishes a protective sanctuary that provides mechanisms of immune escape and chemoresistance but also fosters the acquisition of new genetic abnormalities, generating a vicious cycle that eventually culminates in tumor progression and death. Amongst the myriad of molecules that participate in leukemia-TME interactions, selectins have been recognized to play a substantial role^15,34,35^. Not only do leukemic cells hijack the selectin pathway to disseminate and home into protective niches, but also it has become increasingly evident that selectins provide survival signals both in the circulation as well as in the TME^15,16,21^. Thus, understanding the molecular mechanisms of selectin-ligand binding becomes of utmost importance for the rational design of specific therapies aimed at disrupting these interactions. The success of this strategy can be seen in AML with the E-selectin inhibitor uproleselan (GMI-1271) having entered clinical trials with encouraging results^36^.

We have previously shown that selectin ligands are expressed in a subpopulation of MM cells in vitro and in vivo^25^. These cells are capable of binding E- and P-selectins with high affinity. Binding to E-selectin leads to the development of an aggressive disease in a xenograft mouse model characterized by a complete resistance to bortezomib; importantly this phenotype can be effectively reverted by blocking E-selectin with uproleselan or by sialyltransferase inhibition^25,26^. Binding to P-selectin promotes survival of MM cells by reducing NK cell-mediated cytotoxicity^27^. In this study, we sought to uncover the identity of these selectin ligands and found that the main protein ligand is PSGL-1, a homo-dimeric mucin-like molecule whose importance in MM has been reported^31,37,38^. Indeed, it has been shown that PSGL-1 plays a pivotal role in MM extravasation and in mediating survival signals emanating from the BM microenvironment^31^. Moreover, PSGL-1 gene expression increased with disease progression and the degree of PSGL-1 expression may be used as a prognostic marker in MM^38^. Our study adds another level of complexity as we show that PSGL-1 is modified by SLe^a/x^-related structures only in the Heca452 enriched cells. This observation explains why the Heca452 enriched and the parental isogenic MM cells display a differential binding to E- and P-selectin, despite having the same levels of PSGL-1^25^. This has important implications in the prognostic potential of PSGL-1. Indeed, we clearly show that the function of PSGL-1 cannot be established solely based on its expression levels and a phenotypic as well as biochemical characterization of PSGL-1 may be more appropriate to extrapolate its function and may also correlate better with clinical parameters such as overall survival^38^. Therefore, antibodies that specifically recognize PSGL-1 carrying SLe^a/x^-related modifications or biochemical analysis of PSGL-1 are warranted in the clinic.

We have also performed a functional characterization of the key determinants of E- and P-selectin binding in the Heca452 enriched MM cells employing genetic, pharmacological and biochemical approaches. Using this strategy, we show that while PSGL-1 is required for P-selectin binding, it is dispensable for E-selectin engagement. Indeed, knocking down or neutralizing PSGL-1 using a blocking antibody did not abolish the interactions between MM cells and E-selectin, suggesting that additional protein or lipid ligands compensate for PSGL-1 in E-selectin binding. However, under these conditions we did observe an increase in the rolling velocity of the Heca452 enriched cells on E-selectin, suggesting that the cells establish weak interactions with E-selectin when PSGL-1 is blocked and thus may be less prone to home into the BM. To discriminate the relative contribution of protein and lipid ligands in E-selectin binding, we treated MM cells with pronase, which effectively removed all the SLe^a/x^-related epitopes on glycoprotein carries as shown by Western blot analysis. We observed that pronase treatment did not inhibit rolling and adhesion on E-selectin and did not abolish the Heca452 signal detected by flow cytometry, strongly suggesting that the SLe^a/x^ structures are also displayed on lipid carriers. Glycolipids have been shown to serve as E-selectin ligands on leukocytes^39,40^ and different tumor cells including breast^41^, prostate^42^, colon cancer^43^ and leukemia^44,45^. It should also be noted that pronase treatment decreased the rolling velocity of the Heca452 enriched MM cells, suggesting that in the absence of SLe^a/x^ structures on protein carriers, glycolipids could establish tighter binding to E-selectin. Glycolipids represent short scaffolds compared to proteins^34^, thus, in the presence of protein ligands, glycolipids may make a minor contribution in E-selectin binding. Our results suggest that glycolipids become relevant E-selectin ligands only when fully exposed to the cell surface, a condition that is provided by the pronase treatment. However, further work is needed to determine unambiguously the role and the identity of the glycolipids as E-selectin ligands in MM cells. The Heca452 enriched cells establish an aggressive phenotype in vivo that is completely refractory to bortezomib treatment and is dependent on E-selectin, since it can be reverted by the specific E-selectin inhibitor uproleselan ^25^. As recombinant E-selectin is not able to protect the Heca452 enriched cells from bortezomib in vitro^25^, the current hypothesis is that in vivo, E-selectin promotes homing and retention of the Heca452 enriched MM cells in the BM where they are protected from chemotherapeutic drugs such as bortezomib. In this context, it will be important to establish whether pharmacological inhibition of PSGL-1 could phenocopy the E-selectin inhibitor uproleselan, as our results clearly show that blocking PSGL-1 does not completely suppress E-selectin binding activity. This information has relevant implications in the clinic since blocking PSGL-1 may be insufficient to inhibit E-selectin binding and therefore homing and retention of MM cells into the BM.

Although sialylation is required for E-selectin binding, we observed that this modification is dispensable for the high affinity P-selectin engagement displayed by the Heca452 enriched MM cells. Therefore, the SLe^a/x^-related structures that are recognized by the Heca452 antibody represent *bona fide* markers to predict E-selectin but not P-selectin binding in MM cells. Although SLe^a/x^-related structures are reported to be essential for the binding to all selectins^13,34^, there are examples where sialylation is not required for P-selectin binding, such as in the AML cell line HL-60^46^ and in mouse and human T cells^47,48^. The determinants that allow the Heca452 enriched MM cells to bind with high affinity to P-selectin in the absence of sialylation are presently unknown. However, a fucosylated-related structure may be responsible for this binding since removal of sialic acids by neuraminidase does not abolish fucosylation and could even unmask fucosylated structures on the Heca452 enriched cells, as has been observed in B cell lines^49^. One candidate is CD15, the non-sialylated form of SLe^x^, which is expressed at high levels on the Heca452 enriched cells (data not shown) and can be unmasked by neuraminidase treatment^49^.

Since PSGL-1 mediates survival signals from the BM microenvironment^31^, it will be important to determine whether the sialofucosylated form of PSGL-1 described here retains these functions. Moreover, sialofucosylation of PSGL-1 may also be responsible for the binding of the Heca452 enriched MM cells to platelets, which is P-selectin dependent^27^. Finally, PSGL-1 is an important immune checkpoint regulator in T cells^50,51^ and a ligand for the sialic acid binding immunoglobulin-like lectin (Siglec)-7^52^, immuno-regulatory receptors expressed on macrophages and NK cells. Therefore, sialylation may also modulate these important PSGL-1 functions in MM, contributing to the establishment of an immuno-suppressive TME. Additional work will be needed to address all these different aspects of PSGL-1 biology as it is now clear that it plays an important role in MM.

In this study, we have characterized in detail the binding determinants of the selectin ligands in MM cells, highlighting the differential E- and P-selectin binding requirements. These data have relevant implications for the clinical use of selectin ligands as prognostic markers and therapeutic targets in MM.

## Methods

### Cell lines

The MM1S and RPMI8226 cell lines were from the American Type Culture Collection (ATCC; Manassas, VA). The Heca452 enriched cell lines were generated from the parental lines as previously described^25^. Cells were cultured in RPMI1640 media supplemented with 10% heat inactivated foetal bovine serum (hiFBS), 50 U/ml penicillin and 50 µg/ml streptomycin, all from Merck Millipore (Rahway, NJ). Lenti-X™ 293 T cells were from TaKaRa (Shiga, Japan) and maintained in Dulbecco’s modified eagle media (DMEM) supplemented with 10% hiFBS, 50 U/ml penicillin and 50 µg/ml streptomycin. All reagents are from Merk Millipore unless stated otherwise.

### Immunoprecipitation for mass spectrometry and Western blot analysis

Parental and Heca452 enriched MM1S and RPMI8226 cells (250 × 10^6^) were resuspended in total membrane extraction buffer (0.5 M tris[hydroxymethyl] aminomethane·hydrochloric acid [Tris·HCl] pH 7.7, 0.3 M magnesium chloride [MgCl_2_], 0.1 M Tris·ethylenediamine tetraacetic acid [Tris·EDTA]) supplemented with proteinase and phosphatase inhibitors (1:100) and subjected to three cycles of freezing/thawing in liquid N_2_ (30 s) and water bath at 37 °C (105 s). Nuclei and cell debris were pelleted at 1000$\times$g for 5 min at 4 °C. Supernatant was centrifuged at 100,000$\times$g for 45 min at 4 °C. The pellet containing the membrane-enriched fraction was resuspended in IGEPAL-based buffer (2% IGEPAL, 150 mM sodium chloride [NaCl], 50 mM Tris·HCl, 1 mM EDTA) supplemented with proteinase and phosphatase inhibitors and incubated overnight at 4 °C. Two mg of the resulting membrane-enriched extract were used for each immunoprecipitation (IP) conditions. The samples were first precleared with Dynabeads® M-270 epoxy (ThermoFisher Scientific; Waltham, MS) for 1 h at 4 °C. The precleared samples were then incubated Dynabeads® M-270 epoxy coupled with specific antibodies or isotype controls for 4 h at 4 °C. After incubation, the beads were washed 6 times in IGEPAL-based buffer and proteins were eluted either with 10 mM of recombinant SLe^x^ (30 min at room temperature [RT]) for mass spectrometry or with ammonium hydroxide (NH_4_OH; 0.5 M NH_4_OH, 0.5 mM EDTA, pH 12) for Western blot analysis. The antibodies used for the IP experiments were the following: mouse IgG1 anti-human CD162 (PSGL1) clone KPL-1; mouse IgG1 k isotype control, rat IgM anti-human cutaneous lymphocyte antigen clone Heca452, rat IgM k isotype control, all from BD Biosciences (Franklin Lakes, NJ). For the LC–MS analysis, samples were sent to the University of Bristol proteome facility service, where samples were proteolytically digested in-solution using the DigestPro automated digestion unit followed by LC–MS performed on a Orbitrap Fusion Lumos Mass Spectrometer (ThermoFisher Scientific) equipped with an upstream Ultimate 3000 nano-LC system.

### Lentiviral transduction

To knock down PSGL-1, we used two PSGL-1 shRNAs available from the Merk MISSION® shRNA products with the following target sequences: GCCACCGAATATGAGTACCTA (PSGL-1 shRNA 1; TRCN0000057013) and TCCAAGGCAGGAGGCCATTTA (PSGL-1 shRNA 2; TRCN0000436811). The PSGL-1 shRNAs and the scramble shRNA control were cloned into the pSIH1-H1-Puro shRNA lentiviral expression vector (System Biosciences; Palo Alto, CA) to generate the shRNA transfer vectors.

The PSGL-1 knocked down MM1S^Heca452^ cells were generated by lentiviral transduction. Briefly, the Lenti-X™ 293 T cells were transfected with ready-to-use lentiviral packaging plasmids mix (Cellecta Inc.; Mountain View, CA) and the PSGL-1/scramble control shRNA transfer vectors using the JetPEI (Polyplus-transfection; France) transfection reagent according to the manufacture instructions. The next day, media from the Lenti-X™ 293 T transfected cells was removed and replaced with fresh DMEM media. After 24 h, the media containing the lentiviral particles (lentiviral supernatant) was collected, centrifuged at 500$\times$g for 10 min, filtered through a 0.45 µm syringe filter, mixed with Lenti-X concentrator (TaKaRa) at a ratio of 1 part of Lenti-X concentrator per 3 parts of lentiviral supernatant and incubated overnight at 4 °C. In the meantime, wells from a 24 well plate were coated with 5 µg/cm^2^ of retronectin (TaKaRa) diluted in Dulbecco’s phosphate saline buffer (DPBS; 500 µl/well) and incubated overnight at 4 °C. Next day, the lentiviral supernatant was centrifuged at 1500$\times$g for 45 min at 4 °C. Viral particles were resuspended in 500 µl DPBS, seeded onto retronectin-coated wells and incubated for 6 h at 37 °C. After incubation, wells were washed with 500 µl of DPBS and 500 µl of MM1S^Heca452^ cells were seeded onto the wells at 5 × 10^5^ cells/ml. Cells are expanded for 3–4 days and then passage in the presence of puromycin (1 µM).

### Rolling and adhesion assay under shear stress

The rolling assay was performed in 8 channel microfluidic biochips (Cellix Limited; Dublin, Ireland) using a Mirus Evo Nano Pump (Cellix Limited). The biochip channels were coated with 15 µg/ml of recombinant human E-selectin (PeProtech; London, UK) or P-selectin (Bio-techne; Minneapolis, MN) in Tris·HCl buffer solution (pH 7.4) supplemented with 1 mM CaCl_2_ and incubated overnight at 4 °C. Each channel was blocked with 1% (w/v) of bovine serum albumin (BSA) and incubated at 37 °C for 1 h before the assay. Cells were washed and resuspended in the rolling assay buffer (RPMI1640 without phenol red supplemented with 1% hiFBS, 5 mM 4-(2-hydroxyethyl)-1-piperazineethanesulfonic acid [HEPES] and 1 mM CaCl_2_) at 2 × 10^6^ cells/ml. Eighty µl of cell suspension were loaded onto the channels and the rolling assay was run at 0.5 dyne/cm^2^ at RT. Cells were monitored in 5 different positions along the channel using an A-Plan 10X/0.25 objective (Carl Zeiss Microscopy GmbH; Jena, Germany) of an AX10Vert.A1 Microscope (Carl Zeiss Microscopy GmbH). Thirty frames per position were collected at 0.5 s from each other using a 01 QIClick F-M-12 Mono 12-bit camera (QImaging; Surrey, Canada). Images were acquired using the Vena Flux assay software (Cellix Limited) and the analysis was performed using the Image-Pro Premiere software (Media Cybernetics; Rockville, MD). A rolling cell was defined as a cell travelling a distance corresponding to more than its diameter. The number of cells per position was added to obtain the total number of cells per channel, which was then averaged between the numbers of channels. In some experiments, cells were pre-incubated for 1 h before the rolling assay with a mouse anti-human PSGL-1 blocking antibody (clone 688,102; Bio-techne) or mouse isotype control (clone 11711; Bio-techne) at 10 µg/ml or pre-treated/mock-treated with neuraminidase from *Vibrio cholerae* (1 mU/ml) or pronase (1 mg/ml) for 45 min at RT in serum free RPMI1640 media.

### Flow cytometry analysis

Cells (5 × 10^5^/tube) were collected, washed in DPBS, resuspended in 100 µl stained buffer, stained with PE-conjugated anti-human cutaneous lymphocyte antigen antibody clone Heca452, Alexa-fluor 647-conjugated anti-human PSGL-1 clone KPL-1 (all from BD Biosciences) and incubated for 30 min at RT with gentle rocking. After incubation, cells were washed with DPBS and resuspended in 500 µl staining buffer supplemented with 7-aminoactinomycin D (7-AAD, 1:80; Immunological Science) to exclude dead cells. Cells were acquired on a BD FACSCelesta™ (BD Biosciences). Data were analyzed using the Infinicyt software v 2.0.5.b.007 (Cytognos; Salamanca, Spain).

### Western blot analysis

Cells were lysed in IGEPAL-based buffer supplemented with proteinase and phosphatase inhibitors. Protein concentration in lysates was determined using the bicinchoninic acid (BCA) assay (ThermoFisher Scientific) and an equal amount of protein (20 μg) was resolved on a sodium dodecyl sulphate poly-acrylamide gel electrophoresis (SDS-PAGE), transferred onto a nitrocellulose membrane and blocked for 1 h with 5% (w/v) not fat milk in DPBS. Membranes were probed overnight with anti-human PSGL-1 (clone KPL-1; BD Biosciences), Heca452 (BD Biosciences) and β actin antibodies diluted 1:1000 in 5% (w/v) BSA in DPBS/ 0.05% (v/v) Tween 20 (DPBST). After incubation, primary antibodies were detected with infrared-conjugated goat secondary antibodies (Li-cor; Lincoln, NE) diluted 1:10,000 in 5% not fat milk in DPBS. Blots were images using a Li-cor Odyssey system and analyzed using Image Studio 2.0.38 (Li-cor).

### Statistical analysis

The unpaired one-tailed t test or the two-way ANOVA followed by Sidak’s multiple comparison post-hoc testing were used to determine significance, using P < 0.05 as the cut-off. ***P < 0.001; **P < 0.01; *P < 0.05. GraphPad Prism 6.02 software (Dotmatics; Boston, MA) was used to compute all statistical calculations.

### Supplementary Information

Supplementary Information 1.

Supplementary Information 2.

Supplementary Information 3.

Supplementary Information 4.

Supplementary Information 5.

## Data Availability

The data supporting the findings of this study are contained within the main manuscript and associated Supplementary Information Files. The data are available from the corresponding author upon reasonable request. The mass spectrometry proteomics data have been deposited to the ProteomeXchange Consortium via the PRIDE partner repository with the dataset identifier PXD046178.
