# Supplementary material for: PSGL-1 decorated with sialyl Lewisa/x promotes high affinity binding of myeloma cells to P-selectin but is dispensable for E-selectin engagement
Source: Sci Rep. 2024 Jan 19;14:1756. doi: 10.1038/s41598-024-52212-2 (PMC10798956; doi:10.1038/s41598-024-52212-2)
Supplement: Supplementary file 1 — Supplementary Information 1. [file 41598_2024_52212_MOESM1_ESM.pdf]

PSGL-1 decorated with sialyl Lewis$^{a/x}$ promotes high affinity binding of myeloma cells to P-selectin but is dispensable for E-selectin engagement

Michael O'Dwyer[1], Lucy Kirkham-McCarthy[1], Marina Cerreto[2], Robin Foà[2] and Alessandro Natoni[2*]

[1] Biomedical Sciences, School of Medicine, National University of Ireland Galway, Galway Ireland.

[2] Hematology, Department of Translational and Precision Medicine, Sapienza University, Rome, Italy.

* Correspondence: alessandro.natoni@uniroma1.it. +39 3343373065

The supporting information contains 8 supporting figures and their relative figure legends and 4 supporting tables (provided as 4 separate Excel [.xlsx] files) and their relative legends.

Figure S1

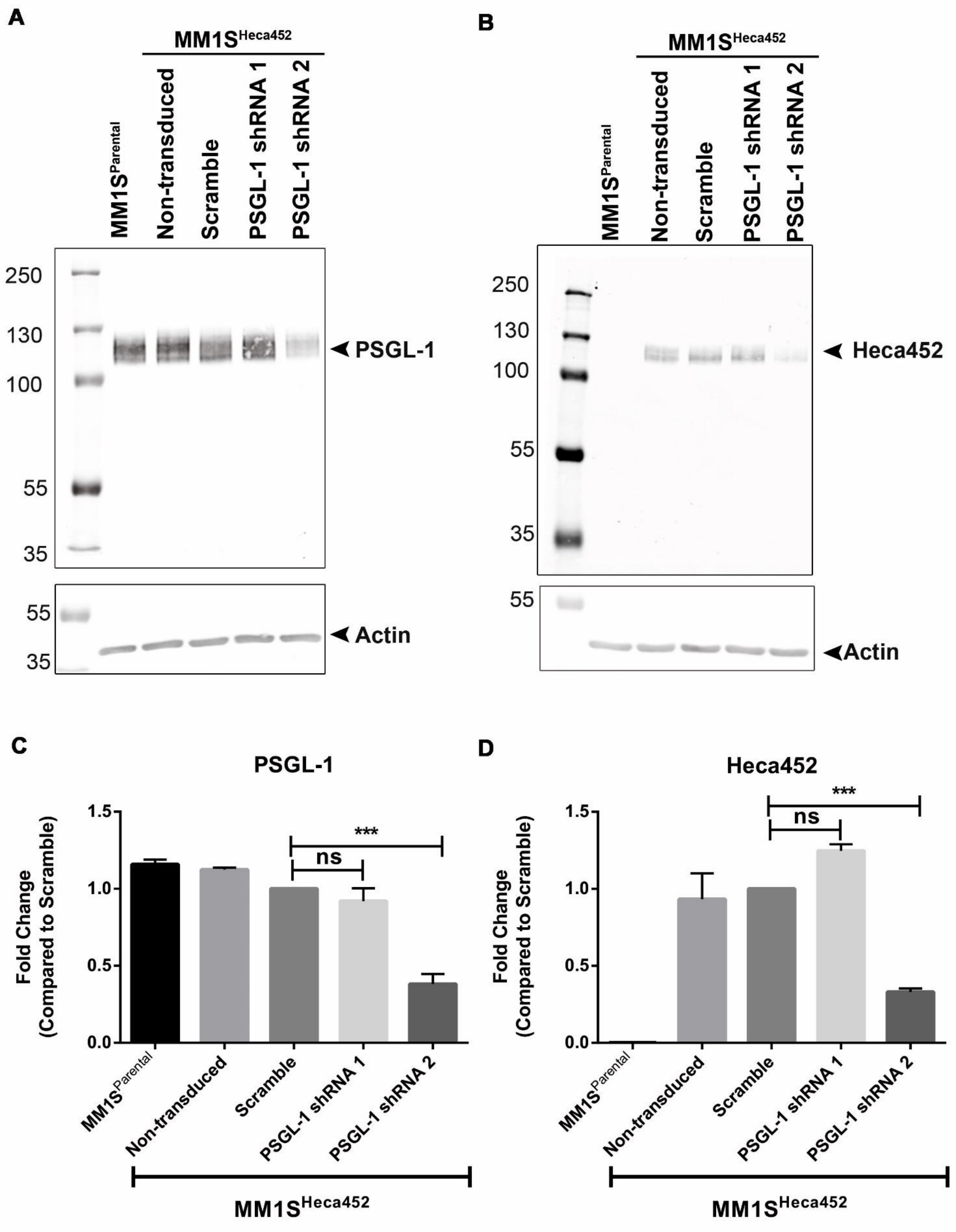
A
MM1S Heca452
MM1S Parental
Non-transduced
Scramble
PSGL-1 shRNA 1
PSGL-1 shRNA 2
250
130
100
55
35
PSGL-1
55
35
Actin
B
MM1S Heca452
MM1S Parental
Non-transduced
Scramble
PSGL-1 shRNA 1
PSGL-1 shRNA 2
250
130
100
55
35
Heca452
55
Actin
C
PSGL-1
Fold Change (Compared to Scramble)
1.5
1.0
0.5
0.0
ns
***
MM1S Parental
Non-transduced
Scramble
PSGL-1 shRNA 1
PSGL-1 shRNA 2
MM1S Heca452
D
Heca452
Fold Change (Compared to Scramble)
1.5
1.0
0.5
0.0
ns
***
MM1S Parental
Non-transduced
Scramble
PSGL-1 shRNA 1
PSGL-1 shRNA 2
MM1S Heca452

Figure S1. PSGL-1 knocked down by shRNAs in MM1S^{Heca452} cells. Cell extracts were prepared from MM1S^{Heca452} cells transduced with control shRNA (scramble) and two shRNA for PSGL-1. MM1S^{Parental} and non-transduced MM1S^{Heca452} cell extracts were used as negative and positive controls for the Heca452 antibody. Cell extracts were subjected to SDS-PAGE, transferred onto a nitrocellulose membrane and blotted for PSGL-1 (A), Heca452 (B) and β-actin, used as loading control. Labels above the blots represent the samples from different cells whereas labels of the right had side of the blots indicate the antibodies used for blotting. Numbers on the left hand side of the blots represent the molecular weight marker. (C, D) Quantification of the PSGL-1 and Heca452 Western blot analysis shown in A and B. The signal intensity obtained from the target bands of each sample was first normalized to the corresponding signal intensity of the actin band and then expressed as fold change relative to the scramble sample. Quantification was carried out using the Image Studio V2.0.38 (Li-cor; Lincoln, NE). Histograms represent the mean + sem of four independent experiments. The two-way ANOVA followed by Sidak's multiple comparison post-hoc testing was used to determine statistical significance. *** $p<0.001$; ns non-significant.

Figure S2

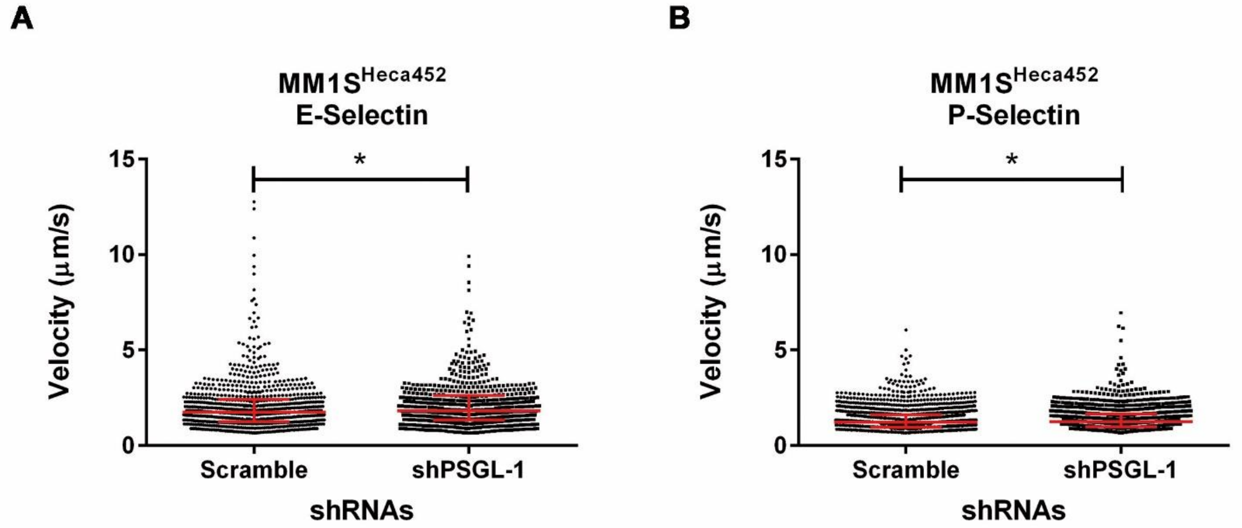

Figure S2. PSGL-1 knocked down increases the rolling velocity of the MM1S^{Heca452} cells on E- and P-selectins. Rolling velocity expressed as µm/s of the MM1S^{Heca452} cells transduced with control (scramble) or specific PSGL-1 (shPSGL-1) shRNAs and perfused on E-selectin (A) and P-selectin (B) coated channels. Bars represent median ± interquartile range. The nonparametric Mann-Whitney test was used to determine statistical significance. * $p<0.01$.

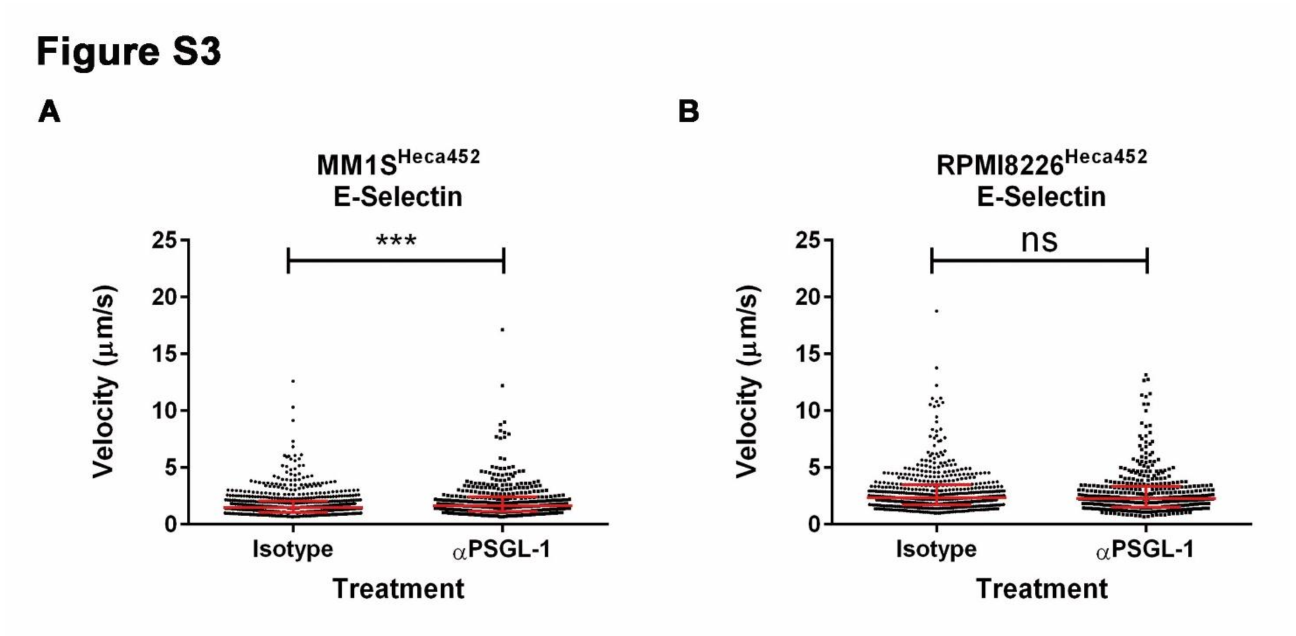

Figure S3. PSGL-1 blocking antibody increases the rolling velocity of the MM1S^{Heca452} cells on E-selectin. Rolling velocity expressed as µm/s of the MM1S^{Heca452} (A) and RPMI8226^{Heca452} (B) cells incubated with an anti-PSGL-1 blocking antibody (αPSGL-1) or matched isotype control (Isotype) and perfused on E-selectin coated channels. Bars represent median ± interquartile range. The nonparametric Mann-Whitney test was used to determine statistical significance. *** $p<0.001$; ns non significant.

Figure S4

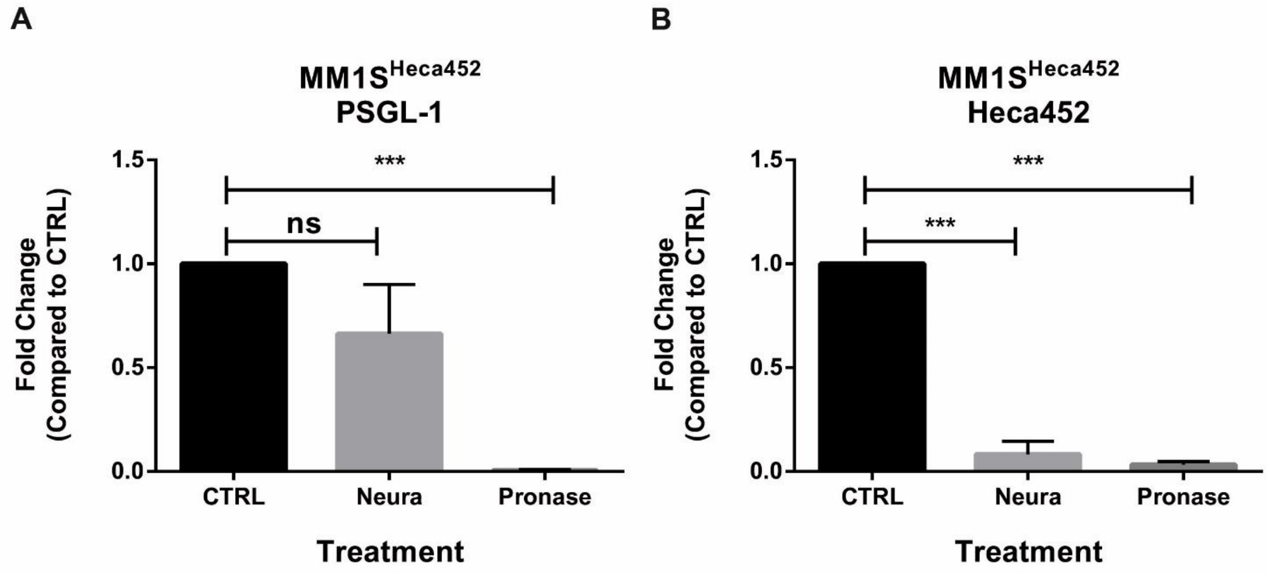

Figure S4. Quantification of the PSGL-1 and Heca452 Western blot analysis shown in Figure 4. The signal intensity obtained from the target bands of each sample was first normalized to the corresponding signal intensity of the actin band and then expressed as fold change relative to the control (CTRL) sample. Quantification was carried out using the Image Studio V2.0.38. Histograms represent the mean + sem of four independent experiments. The two-way ANOVA followed by Sidak's multiple comparison post-hoc testing was used to determine statistical significance. *** $p<0.001$; ns non-significant.

Figure S5

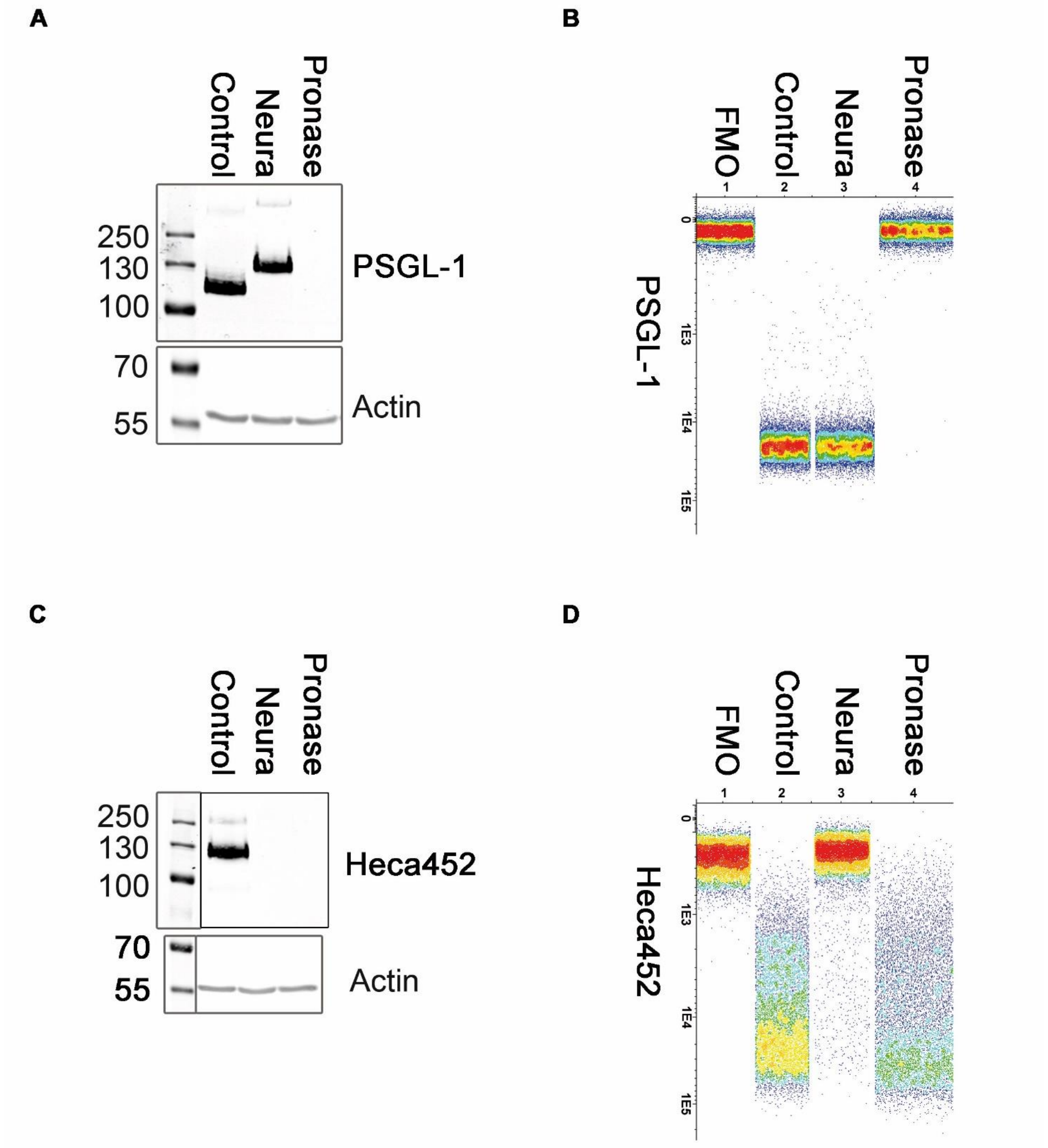

Figure S5. Neuraminidase and pronase treatments differentially affect the levels of PSGL-1 and Heca452 in the RPMI8226^{Heca452} cells. Western blot (A, C) and flow cytometry (B, D) analysis of RPMI8226^{Heca452} cells pre-treated/mock-treated with either neuraminidase (1 mU/ml) or pronase (1 mg/ml) for 45 min at RT. After treatment, cells were either lysed for Western blot analysis or stained with the indicated antibodies and analyzed by flow cytometry. Cell extracts were subjected to SDS-PAGE, transferred onto a nitrocellulose membrane and blotted for PSGL-1, Heca452 and β-actin, used as loading control. Labels above the blots represent the different treatments whereas labels on the right hand side of the blots indicate the antibodies used for blotting. Numbers on the left hand side of the blots represent the

molecular weight marker. The flow cytometry analysis is reported as a band plot generated with the Infinicyt software v 2.0.5.b.007. Labels above the band plots represent the different treatments whereas labels on the left hand side of the band plots indicate the antibody used for the staining. Western blot the flow cytometry analyses depicted in the figure are representative of 3 independent experiments.

Figure S6

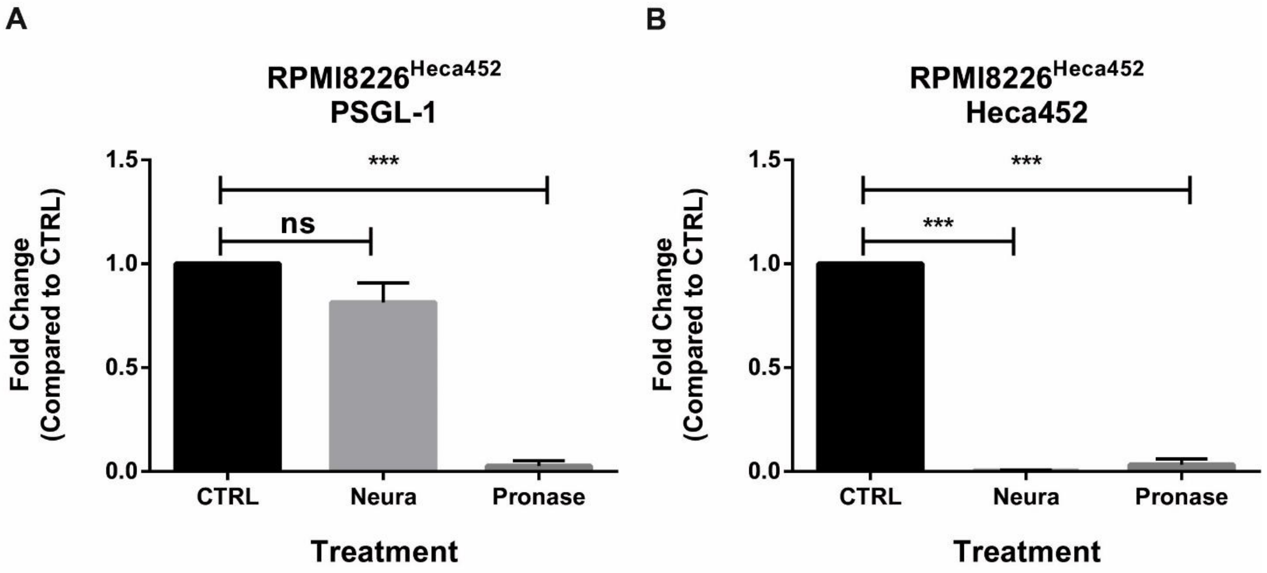

Figure S6. Quantification of the PSGL-1 and Heca452 Western blot analysis shown in Figure S5. The signal intensity obtained from the target bands of each sample was first normalized to the corresponding signal intensity of the actin band and then expressed as fold change relative to the control (CTRL) sample. Quantification was carried out using the Image Studio V2.0.38. Histograms represent the mean + sem of four independent experiments. The two-way ANOVA followed by Sidak's multiple comparison post-hoc testing was used to determine statistical significance. *** $p<0.001$; ns non-significant.

Figure S7

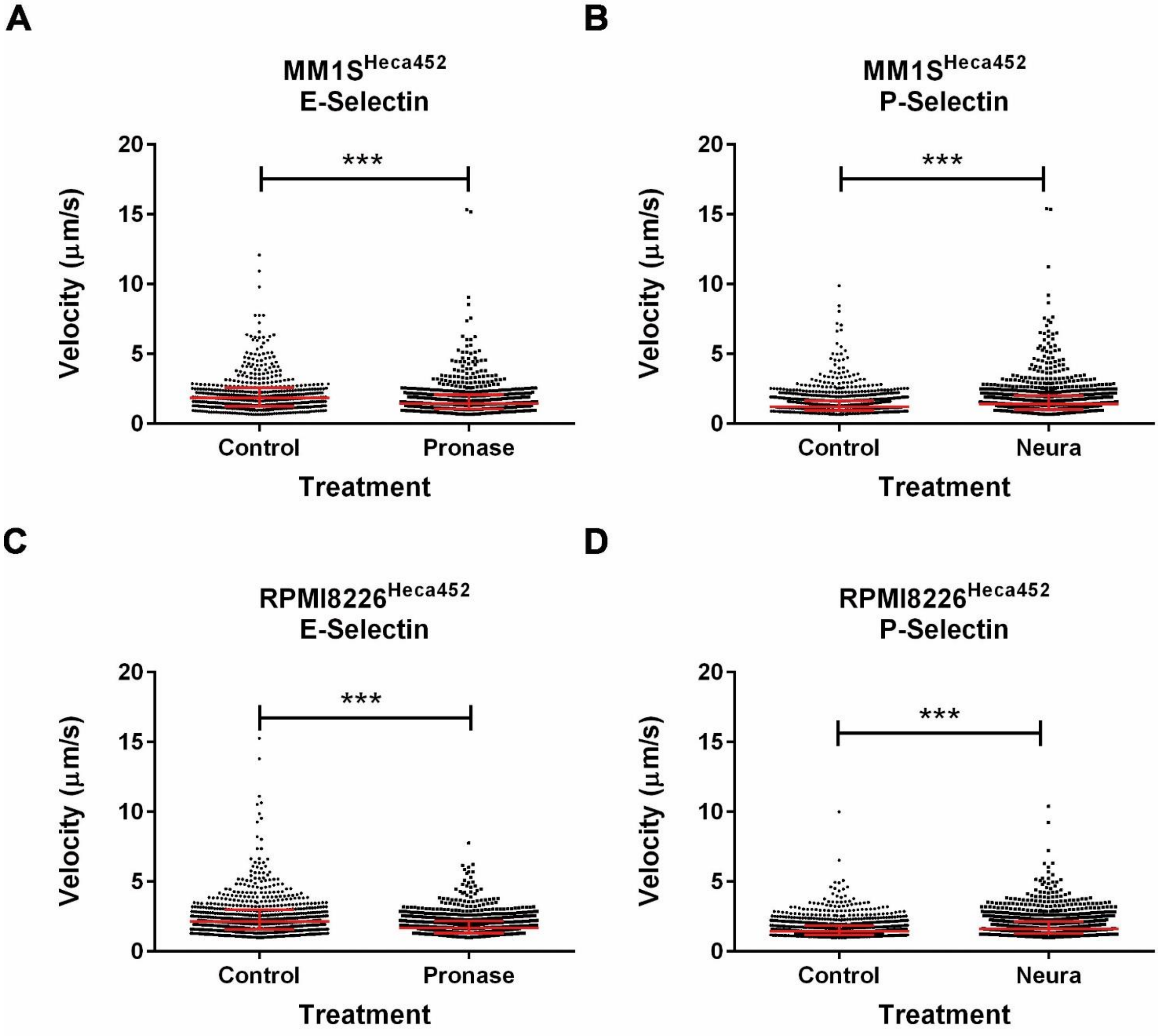

Figure S7. Effects of pronase and neuraminidase treatment on the rolling velocity of MM1S^{Heca452} and RPMI8226^{Heca452} cells. Rolling velocity expressed as µm/s of MM1S^{Heca452} (A, B) and RPMI8226^{Heca452} (C, D) cells pre-treated/mock-treated with pronase (1 mg/ml) (A, C) and neuraminidase (Neura; 1 mU/ml) (B, D) and perfused on E-selectin (A, C) and P-selectin (B, D) coated channels. Bars represent median ± interquartile range. The nonparametric Mann-Whitney test was used to determine statistical significance. *** $p<0.001$.

Figure S8

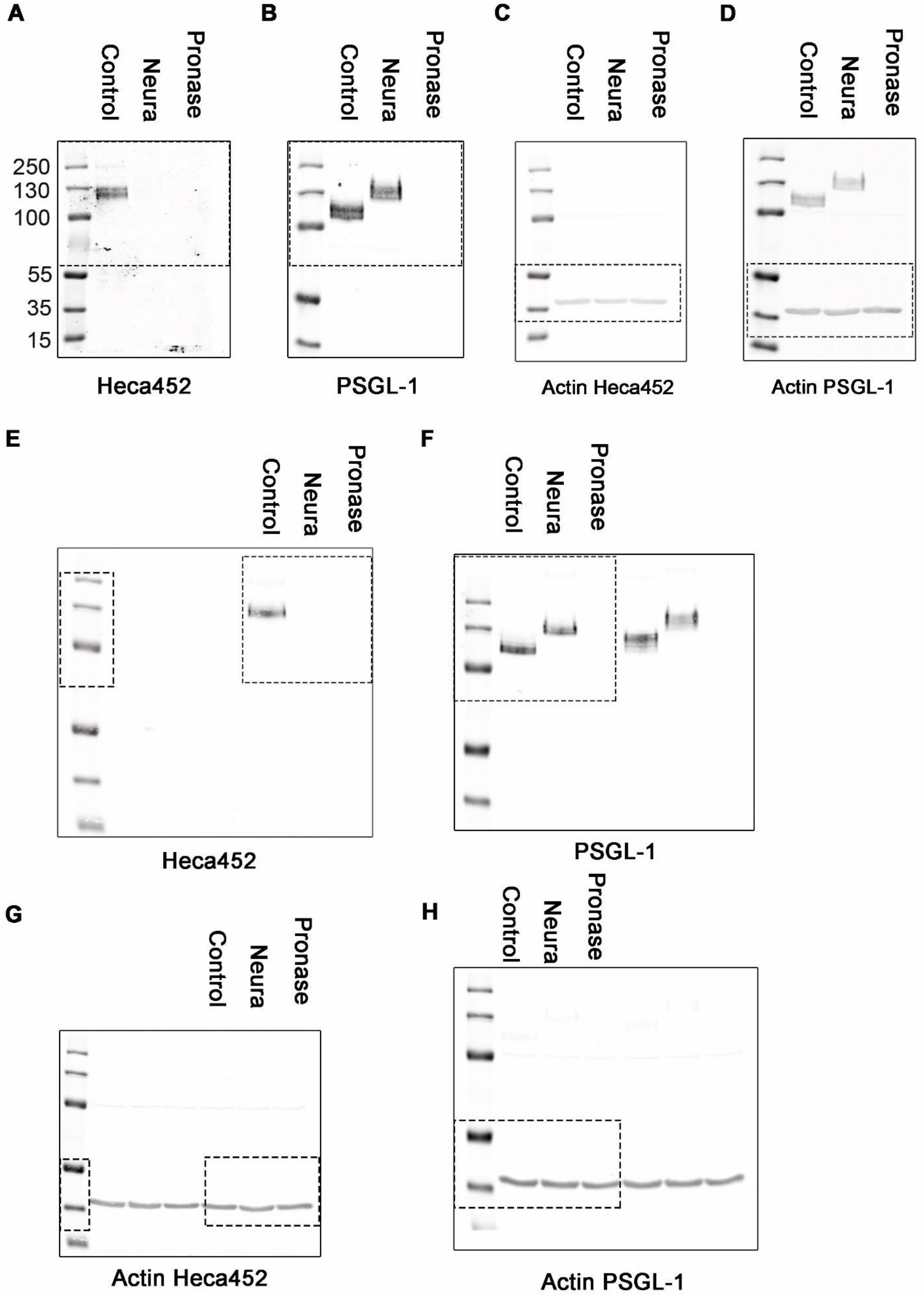
A
B
C
D
Control
Neura
Pronase
250
130
100
55
35
15
Heca452
PSGL-1
Actin Heca452
Actin PSGL-1
E
F
Control
Neura
Pronase
Heca452
PSGL-1
G
H
Control
Neura
Pronase
Actin Heca452
Actin PSGL-1

Figure S8. Uncropped images of the Western blot analysis relative to Figure 4 (A, B, C, D) and Figure S5 (E, F, G, H). The dashed rectangles indicate the area of the image that was cropped. Membranes depicted in A and B represent the same membranes showed in C and D reprobed with the anti-antic antibody respectively. Membranes depicted in E and F represent the same membranes showed in G and H reprobed with the anti-actin antibody respectively.

Figure S9

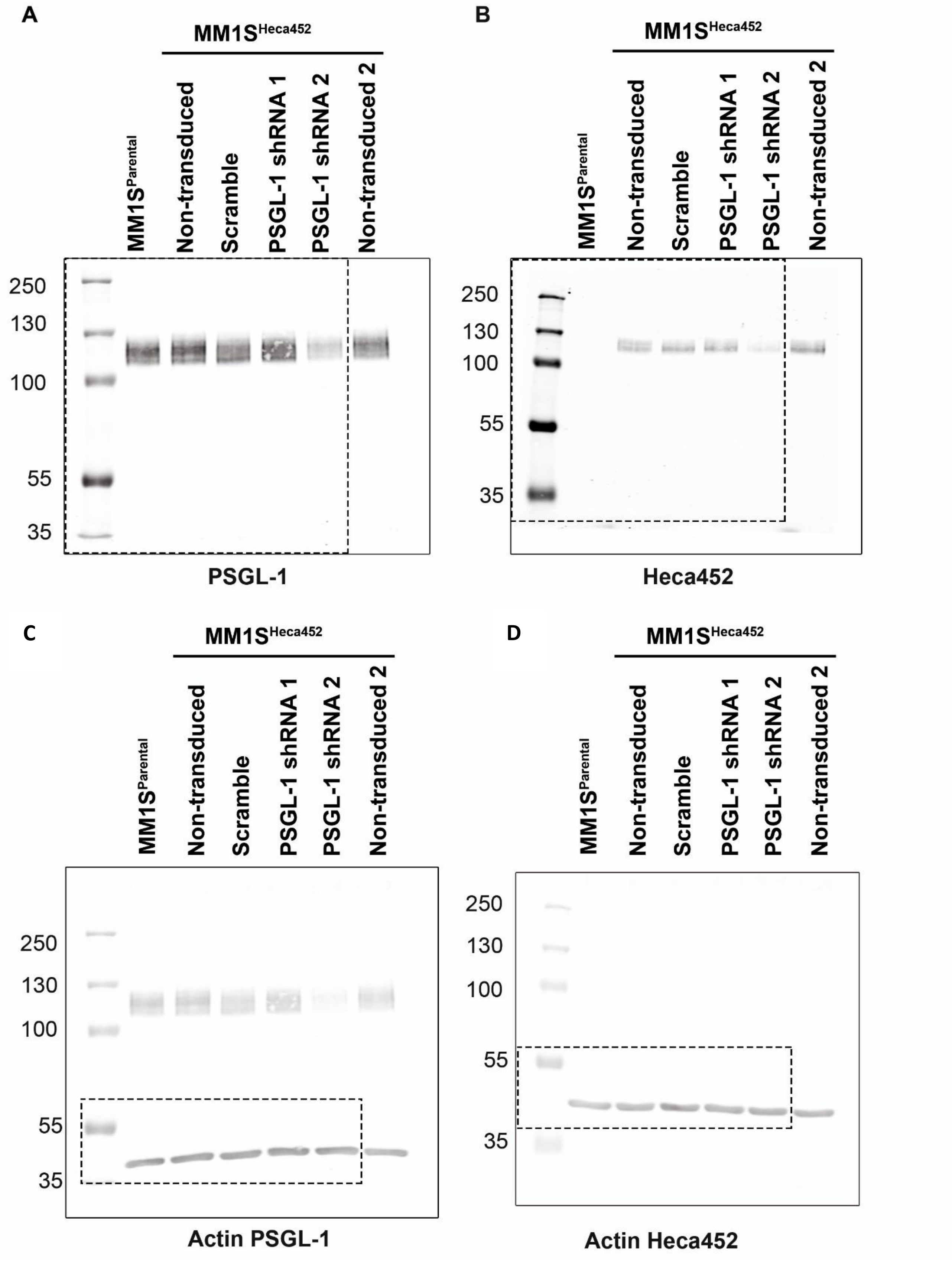
A
MM1SHeca452
MM1SParental
Non-transduced
Scramble
PSGL-1 shRNA 1
PSGL-1 shRNA 2
Non-transduced 2
250
130
100
55
35
PSGL-1
B
MM1SHeca452
MM1SParental
Non-transduced
Scramble
PSGL-1 shRNA 1
PSGL-1 shRNA 2
Non-transduced 2
250
130
100
55
35
Heca452
C
MM1SHeca452
MM1SParental
Non-transduced
Scramble
PSGL-1 shRNA 1
PSGL-1 shRNA 2
Non-transduced 2
250
130
100
55
35
Actin PSGL-1
D
MM1SHeca452
MM1SParental
Non-transduced
Scramble
PSGL-1 shRNA 1
PSGL-1 shRNA 2
Non-transduced 2
250
130
100
55
35
Actin Heca452

Figure S9. Uncropped images of the Western blot analysis relative to Figure S1 (A and B). The dashed rectangles indicate the area of the image that was cropped. Membranes depicted in A and B represent the same membranes showed in C and D reprobed with the anti-actin antibody respectively.

Figure S10

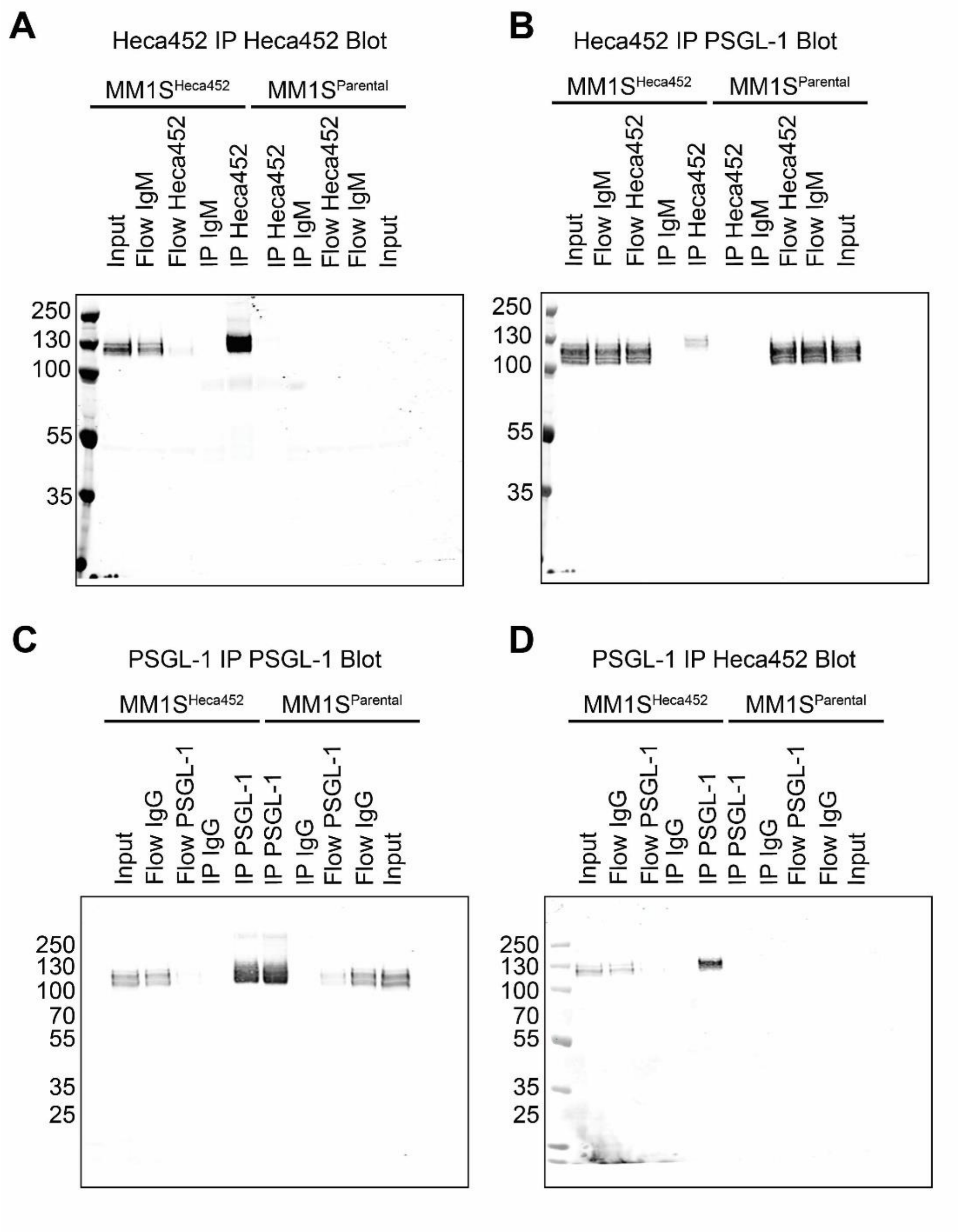

Figure S10. Western blots of Figure 1 showed at high intensity.

Figure S11

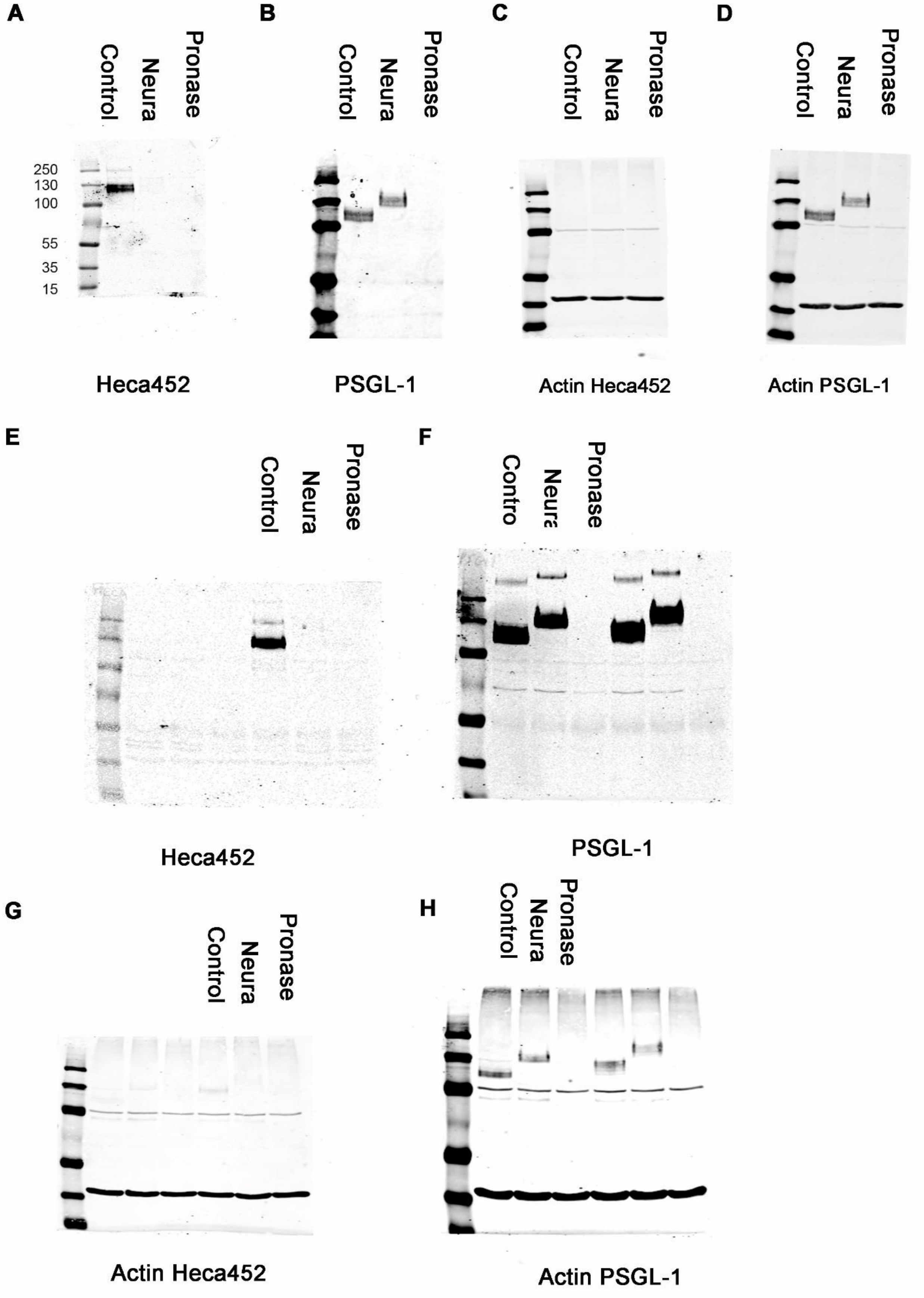
A
Control
Neura
Pronase
250
130
100
55
35
15
Heca452
B
Control
Neura
Pronase
PSGL-1
C
Control
Neura
Pronase
Actin Heca452
D
Control
Neura
Pronase
Actin PSGL-1
E
Control
Neura
Pronase
Heca452
F
Contro
Neura
Pronase
PSGL-1
G
Control
Neura
Pronase
Actin Heca452
H
Control
Neura
Pronase
Actin PSGL-1

Figure S11. Uncropped images of the Western blot analysis of Figure S8 displayed at very high intensity to highlight the edges of the membranes. The membranes depicted in A and B were cut prior antibody hybridization as only three samples were run on the gel. Membranes depicted in A and B represent the same membranes showed in C and D reprobed with the anti-antic antibody respectively. Membranes depicted in E and F represent the same membranes showed in G and H reprobed with the anti-actin antibody respectively.

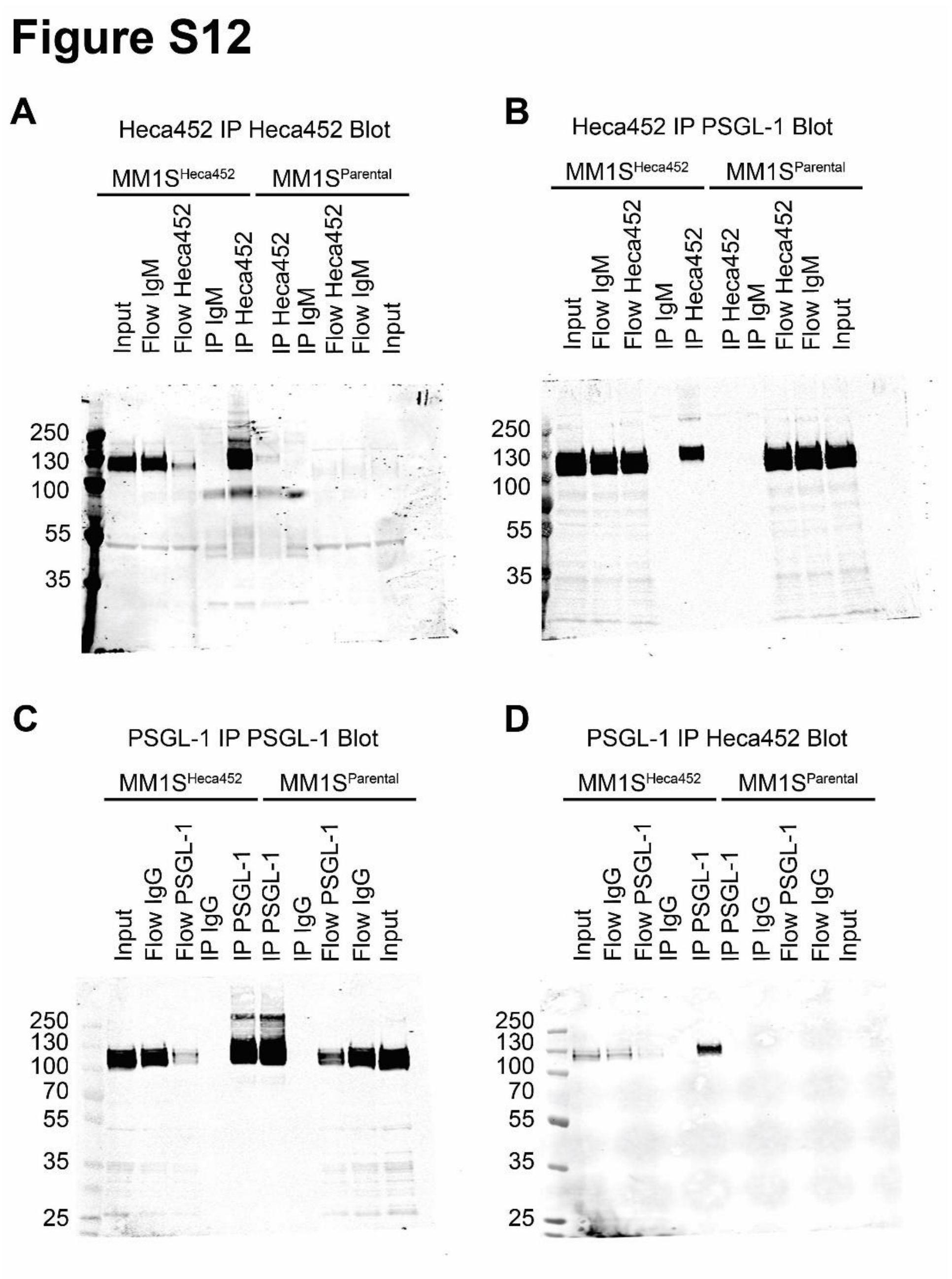

Figure S12. Uncropped images of the Western blot analysis of Figure 1 displayed at very high intensity to show the edges of the membranes. Full length membranes of the Western blots depicted in Figure 1 showed at very high intensity to highlight membrane edges. Membrane depicted in B was cut close to the MW marker on the right.

Table S1. Data set from the Heca452 IP performed on MM1S^{Heca452} membrane-enriched fraction. Proteins identified by LC-MS. Only proteins that were identified by more than 2 peptides are shown. Proteins that were immunoprecipitated by the isotype antibody were excluded. The identified proteins are ranked based on the highest area values. The area value displays the average area of the three unique peptides with the largest peak area. The score value displays the cumulative protein score based on summing the ion scores of the unique peptides identified for that protein. If a peptide was redundantly identified, only the highest-scoring peptide is used. The coverage displays the by default the percentage of the protein sequence covered by identified peptides. The #peptide displays the number of distinct peptide sequences in the protein group. The #PSM displays the total number of identified peptide sequences (peptide spectrum matches) for the protein, including those redundantly identified. The #AAs shows by default the sequence length of the protein. The MW [kDa] and calc.ip display the calculated molecular weight of the protein and the theoretically calculated isoelectric point respectively.

Table S2. Data set from the Heca452 IP performed on MM1S^{parental} membrane-enriched fraction. Proteins identified by LC-MS. Only proteins that were identified by more than 2 peptides are shown. Proteins that were immunoprecipitated by the isotype antibody were excluded. The identified proteins are ranked based on the highest area values. The area value displays the average area of the three unique peptides with the largest peak area. The score value displays the cumulative protein score based on summing the ion scores of the unique peptides identified for that protein. If a peptide was redundantly identified, only the highest-scoring peptide is used. The coverage displays the by default the percentage of the protein sequence covered by identified peptides. The #peptide displays the number of distinct peptide sequences in the protein group. The #PSM displays the total number of identified peptide sequences (peptide spectrum matches) for the protein, including those redundantly identified. The #AAs shows by default the sequence length of the protein. The MW [kDa] and calc.ip display the calculated molecular weight of the protein and the theoretically calculated isoelectric point respectively.

Table S3. Data set from the Heca452 IP performed on RPMI8226^{Heca452} membrane-enriched fraction. Proteins identified by LC-MS. Only proteins that were identified by more than 2 peptides are shown. Proteins that were immunoprecipitated by the isotype antibody were excluded. The identified proteins are ranked based on the highest area values. The area value displays the average area of the three unique peptides with the largest peak area. The score value displays the cumulative protein score based on summing the ion scores of the unique peptides identified for that protein. If a peptide was redundantly identified, only the highest-scoring peptide is used. The coverage displays the by default the percentage of the protein sequence covered by identified peptides. The #peptide displays the number of distinct peptide sequences in the protein group. The #PSM displays the total number of identified peptide sequences (peptide spectrum matches) for the protein, including those redundantly identified. The #AAs shows by default the sequence length of the protein. The MW [kDa] and calc.ip display the calculated molecular weight of the protein and the theoretically calculated isoelectric point respectively.

Table S4. Data set from the Heca452 IP performed on RPMI8226^{Parental} membrane-enriched fraction. Proteins identified by LC-MS. Only proteins that were identified by more than 2 peptides are shown. Proteins that were immunoprecipitated by the isotype antibody were excluded. The identified proteins are ranked based on the highest area values. The area value displays the average area of the three unique peptides with the largest peak area. The score value displays the cumulative protein score based on

summing the ion scores of the unique peptides identified for that protein. If a peptide was redundantly identified, only the highest-scoring peptide is used. The coverage displays the by default the percentage of the protein sequence covered by identified peptides. The #peptide displays the number of distinct peptide sequences in the protein group. The #PSM displays the total number of identified peptide sequences (peptide spectrum matches) for the protein, including those redundantly identified. The #AAs shows by default the sequence length of the protein. The MW [kDa] and calc.ip display the calculated molecular weight of the protein and the theoretically calculated isoelectric point respectively.
